# Halogen-Containing Drugs in 2025: A Record Year for the Therapeutic Use and Synthesis of FDA-Approved Small Molecules

**DOI:** 10.3390/biom16030381

**Published:** 2026-03-03

**Authors:** Davide Benedetto Tiz, Marco D’Alì, Nunzio Iraci, Claudio Santi, Luca Sancineto

**Affiliations:** 1 Laboratory of Molecular Logic Gates, Department of Chemistry, Faculty of Science, University of Malta, MSD 2080 Msida, Malta; 2 Department of Chemical, Biological, Pharmaceutical, and Environmental Sciences, University of Messina, Viale Ferdinando Stagno d’Alcontres 31, 98166 Messina, Italy; 3 Group of Catalysis and Organic Green Chemistry, Department of Pharmaceutical Sciences, University of Perugia, Via del Liceo 1, 06123 Perugia, Italy

**Keywords:** halogens, approved drugs, FDA, synthesis, medicinal chemistry

## Abstract

Halogens, particularly fluorine, chlorine, and bromine, play a pivotal role in modern drug discovery and development. Their incorporation into drug molecules significantly influences physicochemical properties, including lipophilicity, metabolic stability, and target binding affinity. Fluorine, the most commonly used halogen, enhances bioavailability and receptor interactions, as seen in several blockbuster drugs. Chlorine and bromine contribute to hydrophobic interactions and modulate pharmacokinetics, while iodine is less frequently utilized due to its larger atomic size and reactivity. The strategic placement of halogens in drug scaffolds has led to the success of numerous FDA-approved pharmaceuticals across therapeutic areas, including oncology, infectious diseases, and central nervous system disorders. This review explores the structure–activity relationships (SAR) of halogen-containing drugs, highlighting recent approvals (2025), their synthesis (with yields, when available), therapeutic use, and, when experimentally available, the interaction with their biological target macromolecules.

## 1. Introduction

In our previous works [1,2,3,4] and especially in [1], we have highlighted the emergent role of halogens and, in particular, of fluorine and chlorine in the preparation of drugs for the treatment of several diseases, such as viral infections, several types of cancer, cardiovascular disease, multiple sclerosis, migraine, and inflammatory diseases such as vasculitis. Talking about numbers, we showed that 14 of the 50 molecules approved by the FDA in 2021 contained halogens. In 2022, only six drugs approved contained halogens [5]. In 2023, the number rose to 14 [5]. More recently, namely in 2024, the U.S. Food and Drug Administration (FDA) approved 50 new drugs, of which 16 contain halogen atoms such as fluorine and chlorine. Therefore, it is evident that halogens play a significant role in drug discovery and development due to their impact on the pharmacological properties of compounds [5].

**Figure 1:**
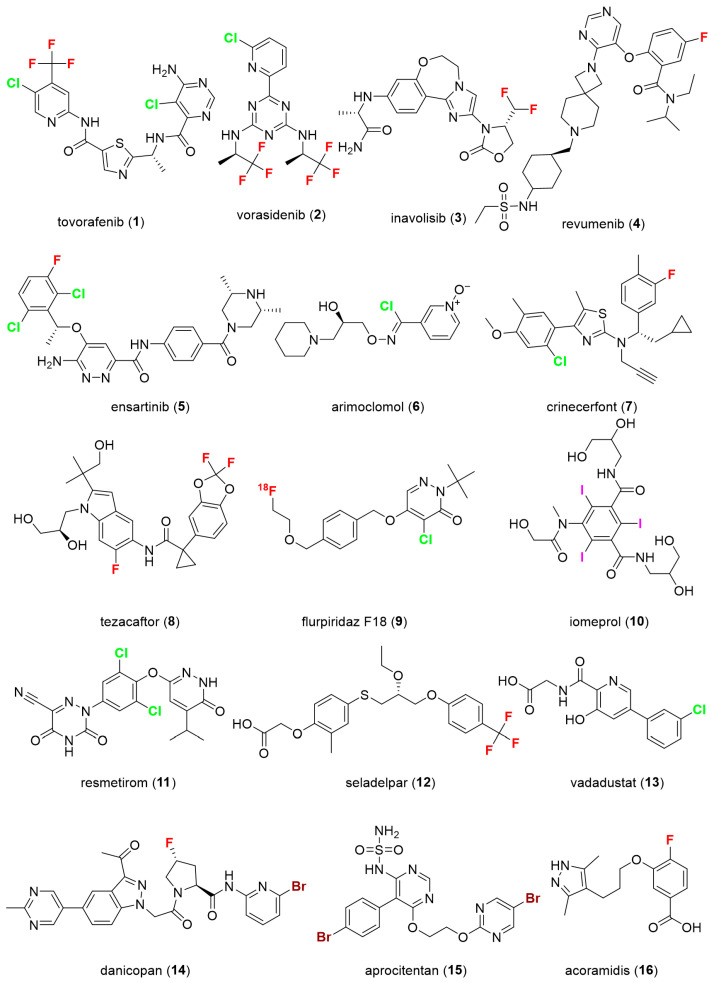
FDA-approved drugs in 2024 containing halogens.

The review by Ali et al. [5] highlights the importance of fluorine and chlorine in medicinal chemistry, discussing their properties and how they influence drug profiles. The authors provided detailed information on each halogen-containing drug approved in 2024, including: trade name, active ingredients, route of administration, approval date, indication, and mode of action. The 16 halogen-containing approved drugs in 2024 are shown in Figure 1: tovorafenib **1** (fluorine-based), vorasidenib **2** (fluorine- and chlorine-based), inavolisib **3** (fluorine-based), revumenib **4** (fluorine-based), ensartinib **5** (fluorine and chlorine-based), arimoclomol **6** (chlorine-based), crinecerfont **7** (fluorine and chlorine-based), tezacaftor **8** (fluorine-based), flurpiridaz F18 **9** (fluorine- and chlorine-based), iomeprol **10** (iodine-based), resmetirom **11** (chlorine-based), seladelpar **12** (fluorine-based), vadadustat **13** (chlorine-based), danicopan **14** (fluorine- and bromine-based), aprocitentan **15** (bromine-based), acoramidis **16** (fluorine-based) [6]. Importantly, the majority of approved drugs contain at least covalently bound fluorine, which is often combined with another halogen (most often chlorine or bromine). Only one drug, iomeprol, contains iodine. Among fluorine-containing drugs, trifluoromethyl groups are encountered in three drugs (**1**, **2**, and **12**). Aromatic single-atom fluorine is present in five drugs (**4**, **5**, **7**, **8**, and **16**). Aliphatic-type fluorine is contained in **3**, **8**, **9**, and **14**. Moreover, **9** is a novel positron emission tomography (PET) myocardial perfusion imaging tracer [7].

**Figure 2:**
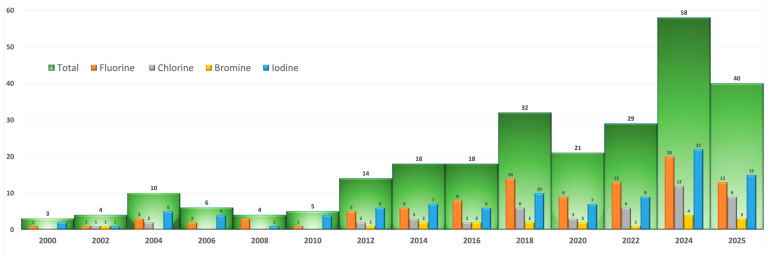
Bar charts obtained from PubMed data using the general terms “halogen approved drugs”, “fluorine approved drugs”, “bromine approved drugs”, “chlorine approved drugs”, and “iodine approved drugs” in the period of 2000–2025. Data assessed on 30 May 2025.

This trend, that is, the majority of approved drugs contain fluorine or chlorine, is confirmed by our analysis presented in Figure 2. The bar charts were created using data retrieved from PubMed using the general terms “fluorine approved drugs” [8], “bromine approved drugs” [9], “chlorine approved drugs” [10], and “iodine approved drugs” [11] in the period of 2000–2025. The arithmetic sum of each halogen contribution is reported. It appears that fluorine and chlorine are dominating in terms of number. Iodine also yielded a high number of hits. This could be explained by the fact that many advanced radioactive iodine therapeutics are in the pipeline.

Ali’s [5], our [1] work, and many other comprehensive reviews [12,13] have previously shown that the careful addition of fluorine or fluorine-containing groups (e.g., CHF_2_, CF_3_, OCF_3_) to electron-rich heteroaromatic and aromatic rings or olefins that are vulnerable to oxidation may result in greater metabolic stability. Fluorine can increase metabolic stability by acting as an isostere of carbonyl groups, eliminating reductive reactions [14]. Moving to physicochemical parameters, adding fluorine to aryl or vinyl moieties increases their lipophilicity. In contrast, adding fluorine to an aliphatic system diminishes lipophilicity [15].

Chlorine can serve as a bioisostere for several functions, including halides (F, Br, I), monovalent substituents (OH, SH), and pseudohalides (CF_3_ and CN) [16]. Chlorine’s similar physicochemical qualities allow it to operate as a bioisostere of methyl groups, improving metabolic stability in vivo [5]. Chlorine is a more effective halogen bond donor than fluorine due to its bigger size [17]. According to certain literature, the addition of bromine can increase the affinity and binding strength to its target via halogen bonds [18]. While bromine has potential properties, other studies suggest it can have negative consequences on the human body [19].

Iodine isotopes have potential for drug discovery and development, but their instability in the body due to weak bonds with organic molecules limits their usage [5]. Interestingly, one iodine-containing drug (iomeprol **10**) was approved in 2024 as a contrast agent.

In the last year (2025), several halogen-containing small molecules (MW < 600 Da) were approved by the FDA. In the next sections, the novel drugs (“novel” drugs are new drugs never before approved or marketed in the U.S. [20]) will be presented according to their date of approval. For each molecule, a notable synthetic pathway (those from the inventors will be privileged) will be given together with the therapeutic indication.

## 2. Halogen-Containing Drugs Approved by the FDA in 2025

2025 appeared to be a promising year for drug development. The focus on novel therapies for unmet medical needs, including cancer treatments, vaccines, and treatments for rare diseases, yielded several new drugs, including 17 halogen-containing drugs.

The following sections will describe the synthetic pathways of approved halogenated small molecules and, when experimental structures are available, will report an analysis of their target–ligand interactions, performed with the aid of software such as PLIP (Protein-Ligand Interaction Profiler, version 2.3.0) [21], Maestro (version 14.4.138) [22], and PyMOL (version 3.2.0) [23].

### 2.1. Suzetrigine (***17***)

**Scheme 1:**
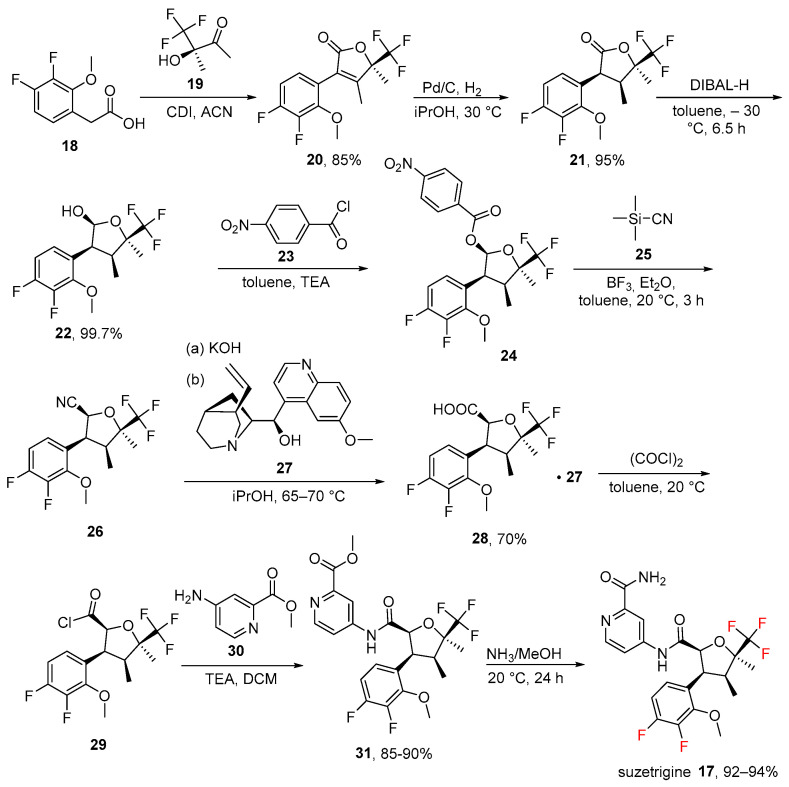
Preparation of suzetrigine (**17**).

Approved on 30 January 2025 [20] and developed by Vertex Pharmaceuticals, Boston, MA, USA, suzetrigine (**17**, Journavx^®^) is a potent and selective inhibitor of the NaV1.8 pain signal for the treatment of moderate-to-severe pain [24]. Voltage-gated sodium channel 1.8 (NaV1.8) is a genetically and pharmacologically verified pain target that is exclusively expressed in peripheral pain-sensing neurons and not in the central nervous system (CNS) [24]. This approval was reported as “an important public health milestone in acute pain management” by the FDA [25]. Unlike opioids, this medicine does not cause euphoria or excitation in the brain, which eliminates the risk of addiction [26]. Suzetrigine’s approval emphasizes that Nav1.8 targeting may be a crucial tactic for managing moderate to severe acute pain [26]. At the moment, the number of Nav1.8 inhibitors in clinical trials is still low. With an IC_50_ of 0.7 nM and more than 31,000-fold selectivity against other NaV isoforms, VX-548 is a superbly selective inhibitor of NaV1.8. Important off-target effects on NaV1.4 (muscle contraction) and NaV1.5 (cardiac rhythm) are prevented by this selectivity [27]. Its structure and synthesis [28,29] (taken from its patent assignee and developers, Vertex Pharmaceuticals) is shown in Scheme 1. It originates from fluorine-based carboxylic acid **18** that is condensed with alcohol **19** via 1,1′-carbonyldiimidazole (CDI) in acetonitrile (ACN) to give unsaturated lactone **20** (85% yield). The double bond in **20** was reduced by catalytic hydrogenation in isopropanol (*i*PrOH) to give lactone **21** (95%). Carbonyl of ester was reduced by diisobutylaluminum hydride (DIBAL-H) to give alcohol **22** (99.7%), which was protected by using *p*-nitrobenzoyl chloride **23** in basic conditions (triethylamine, TEA) to give **24** (yields not disclosed in the primary patent literature). This underwent cyanosilylation via trimethylsilyl cyanide ((CH_3_)_3_Si-C≡N, **25**) to give nitrile **26** (yields not disclosed in the primary patent literature). Hydrolysis (potassium hydroxide, KOH) of nitrile, accompanied by diastereomeric resolution by quinine (**27**), afforded carboxylic acid **28** (70%). Conversion to acyl chloride **29** (yield not reported) mediated by oxalyl chloride (COCl)_2_ was followed by reaction with nucleophilic aniline **30** using TEA in dichloromethane (DCM) that gave amide **31** (85–90%). Eventually, treatment with ammonia in methanol (NH_3_/MeOH) solution resulted in suzetrigine in excellent yield (92–94%, yield range as reported by the inventors). From a recent pharmacology study [24,30] it appeared that suzetrigine is highly selective for NaV1.8 (≥31,000-fold vs. other NaV subtypes). Therefore, the halogen substitutions could contribute to that selectivity by optimizing interactions specific to NaV1.8.

Non-opioid analgesics, particularly NSAIDs, have been linked to gastrointestinal (GI) adverse effects, including dyspepsia, nausea, vomiting, and peptic ulcer, among others [30]. Some selected physicochemical parameters for suzetrigine were *LogP* (logarithm of the partition coefficient; it measures the lipophilicity of a compound based on its distribution between octanol and water when the molecule is in its neutral form): 2.42; tPSA (topological polar surface area): 103.01; and LogS (logarithm of aqueous solubility): −5.021. Its non-halogenated counterpart displayed lower *LogP*, the same tPSA, and higher solubility in water (*LogP*: 1.46, tPSA: 103.01, LogS: −3.55). In essence, this comparison is an example of a strategic trade-off: sacrificing solubility to potentially gain improvements in potency, metabolic stability, or cell permeability. The physicochemical parameters (*LogP*, tPSA, and LogS) were calculated by us using ChemDraw Professional 16.0.1 [31].

### 2.2. Mirdametinib (***32***)

Approved on 11 February 2025, mirdametinib (**32**, Gomekli^®^, Springworks Therapeutics Inc., Stamford, CT, USA) is a kinase inhibitor for adult and pediatric patients 2 years of age and older with neurofibromatosis type 1 (NF1) who have symptomatic plexiform neurofibromas (PN) not amenable to complete resection [32]. It is selectively targeting mitogen-activated protein kinase (MEK), an integral kinase within the mitogen-activated protein kinase 1/2 (MAPK) family [33]. Inhibitors such as mirdametinib show promise by crossing the blood–brain barrier (BBB) [34]. It is orally administered, making it convenient for patients [35]. Common side effects include rash, diarrhea, fatigue, and nausea [32]. Chemically, it is a hydroxamic acid ester produced from benzohydroxamic acid, with the hydroxamic acid group converted into a 2,3-dihydroxypropyl ester. The chemical structure contains a 2-fluoro-4-iodophenylamino group at position 2 of the benzene ring, with fluorine substitutions at positions 3 and 4, a secondary amine linker, a difluorobenzene, and an organoiodine benzene ring with strong MAPK kinase inhibitory capabilities [36].

**Scheme 2:**
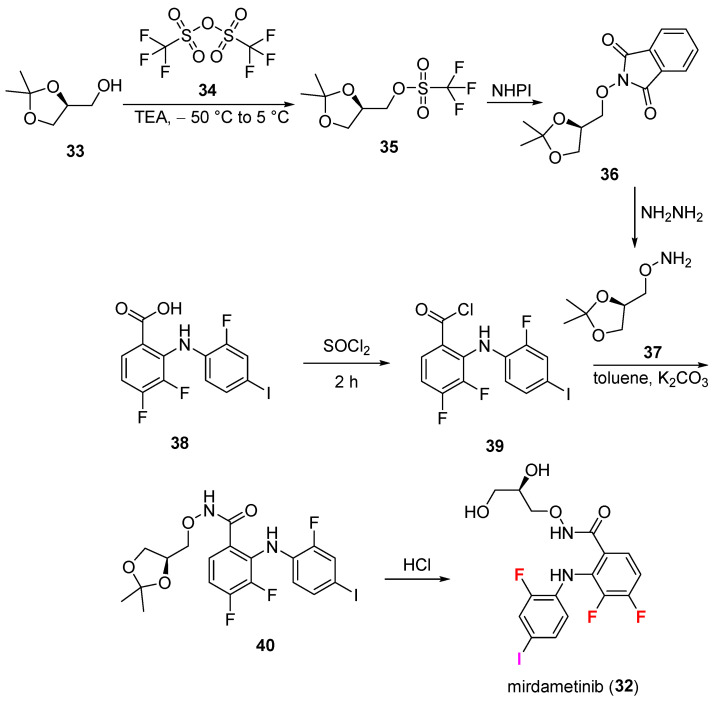
Preparation of mirdametinib (**32**).

Its synthesis [37] (Scheme 2) originates from hydroxylamine **37** that is prepared from alcohol **33**, which is protected with trifluoromethanesulfonic anhydride 34 to give triflate **35**. This was reacted with *N*-hydroxyphthalimide to give **36**. Hydroxylamine **37** was obtained from **36** via hydrazinolysis. The second part of the synthesis involves the highly halogenated biphenyl carboxylic acid **38**, which was activated to acyl chloride **39** by using thionyl chloride (SOCl_2_), followed by treatment with previously obtained **37** in the presence of potassium carbonate (K_2_CO_3_) in toluene to give acetonide **40**. This was converted to product mirdametinib (**32**) in the presence of hydrochloric acid (HCl) to deprotect the acetonide to vicinal diol. Overall yield was 58%. The pharmacokinetics support that **32** has improved metabolic stability compared to earlier MEK inhibitors [38]. The majority of the medication is excreted unaltered, with around 68% in urine and 27% in feces [39].

Treatment-related side effects observed with mirdametinib include dermatitis acneiform, diarrhea, nausea, vomiting, fatigue, dry skin, alopecia, decreased ejection fraction, increased blood creatinine phosphokinase, dizziness, pruritus, constipation, rash, abdominal pain, acne, dry mouth, peripheral edema, skin pain, urticaria, blurred vision, eczema, stomatitis, increased weight, hair color change, hair texture abnormality, extremity pain, paronychia and headache, among others [36].

**Figure 3:**
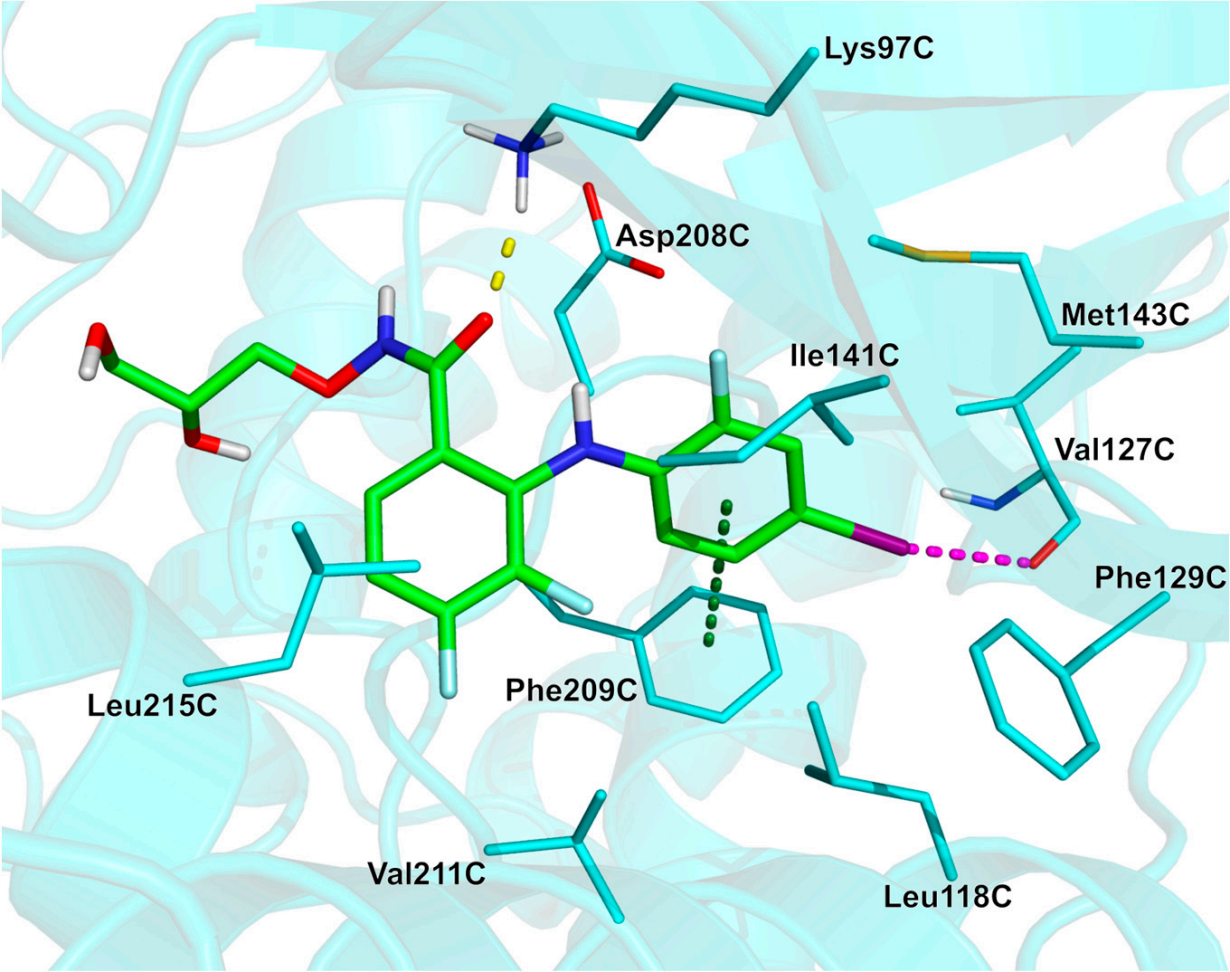
Experimental bound conformation of mirdametinib (PDB: 7JUU [40], crystal structure of KSR2:MEK1 in complex with AMP-PNP and allosteric MEK inhibitor mirdametinib). Mirdametinib is depicted as thick green sticks, MEK1 is represented by cyan cartoons and thin sticks. H-bond is represented by a yellow dashed line, halogen bond by a magenta dashed line, and π-π stacking interaction by a dark green dashed line.

Analysis of the experimentally solved KSR2:MEK1/mirdametinib complex (PDB: 7JUU [40]; crystal structure of human KSR2 (kinase suppressor of RAS 2) and MEK1 (MAPK/ERK Kinase 1), KSR2:MEK1 in complex with AMP-PNP, and allosteric MEK inhibitor mirdametinib) suggest that the diarylamine moiety is accommodated into a pocket lined by residues Lys97C, Leu118C, Val127C, Phe129C, Ile141C, Met143C, Asp208C, Phe209C, Val211C, and Leu215C, establishing hydrophobic interactions with Leu118C, Ile141C and Leu215C. In addition, a H-bond interaction is found between the ligand carbonyl oxygen and the side chain of residue Lys97C, and a π-π stacking interaction is formed between the 2-fluoro-4-iodobenzene ring and the phenyl ring of Phe209C. Of note, a relevant halogen bond interaction is identified between the backbone carbonyl oxygen of Val127C and the iodine atom of the 2-fluoro-4-iodobenzene moiety. This interaction is critical for the stability of the drug–enzyme complex and for the inhibitory activity of the mirdametinib [41] (see Figure 3, PDB: 7JUU [40], crystal structure of KSR2:MEK1 in complex with AMP-PNP and allosteric MEK inhibitor mirdametinib).

Physicochemical parameters of mirdametinib (*LogP*: 3.61, tPSA: 90.82, LogS: –5.173) compared to the non-halogenated derivative (*LogP*: 1.78, tPSA: 90.82, LogS: –3.332) showed that iodine especially contributed to the “lipophilicity” of the drug.

### 2.3. Avutometinib (***41***) and Defactinib (***42***)

**Figure 4:**
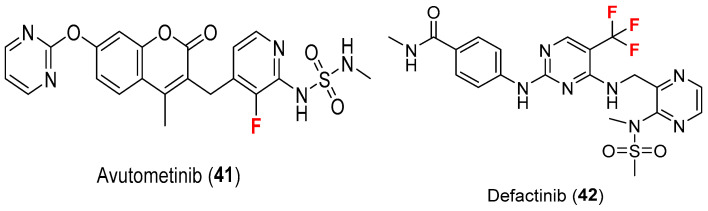
Structures of avutometinib (**41**) and defactinib (**42**).

Both approved on 8 May 2025, the combination of avutometinib (**41**) and defactinib (**42**), also known as Avmapki^®^ Fakzynja^®^ Co-Pack, consists of two fluorine-containing drugs (Figure 4) for the indication KRAS-mutated recurrent low-grade serous ovarian cancer (LGSOC) after prior systemic therapy [42].

Avutometinib (also known as VS-6766), developed by Chugai Pharmaceutical Co., Ltd. Chūō-ku, Tokyo, Japan, in collaboration with Roche, Basel, Switzerland [43], is a small-molecule inhibitor that uniquely targets both mitogen-activated protein kinase (MEK) and rapidly accelerated fibrosarcoma (RAF) kinases, disrupting a critical signaling pathway often dysregulated in cancer [43]. Unlike traditional MEK inhibitors, avutometinib prevents the phosphorylation of MEK by RAF, effectively shutting down the RAF–MEK–ERK cascade at two key nodes [44]. This dual inhibition enhances antitumor activity and reduces compensatory feedback mechanisms that frequently lead to drug resistance [45].

The ATP-competitive focal adhesion kinase (FAK) inhibitor defactinib, often referred to as VS-6063, was developed by Pfizer and is currently owned by Verastem [46]. FAK is frequently overexpressed or activated in various cancers, contributing to tumor progression, metastasis, and resistance to therapy [47]. By targeting FAK, defactinib disrupts tumor–stroma interactions and impairs the cancer cells’ ability to survive under stress or evade immune detection [48].

The most common adverse effects of the drug combination are diarrhea (8%), anemia (5%), and dermatitis acneiform (4%) [42]. Pyridine is well absorbed from the gastrointestinal tract in mammals and undergoes extensive metabolism by C- and *N*-oxidation and by *N*-methylation, a likely pathway in avutometinib [49].

**Scheme 3:**
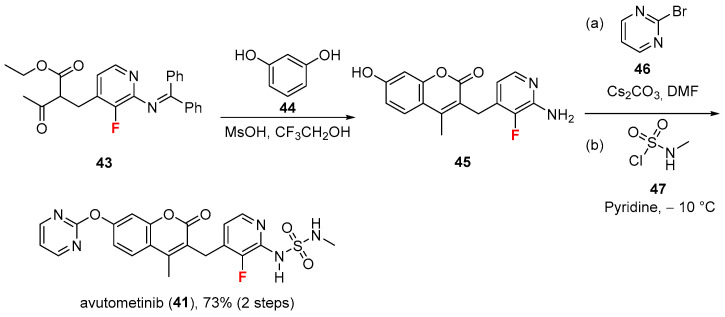
Preparation of avutometinib (**41**).

The synthesis of avutometinib (**41**) [50] (Scheme 3) proceeds via mixing a fluorinated pyridine derivative **43** with resorcinol **44** under acidic (methanesulfonic acid, MsOH) conditions in trifluoroethanol (CF_3_CH_2_OH) to give coumarin derivative **45**. The following SNAR on the phenolic oxygen (using 2-bromopyrimidine **46** as electrophile in cesium carbonate, Cs_2_CO_3_) and the acylation using *N*-methylsulfamoyl chloride **47**, pyridine as a base, and *N*,*N*-dimethylformamide (DMF) as solvent led to avutometinib (**41**) in excellent yield (73%, two steps).

**Figure 5:**
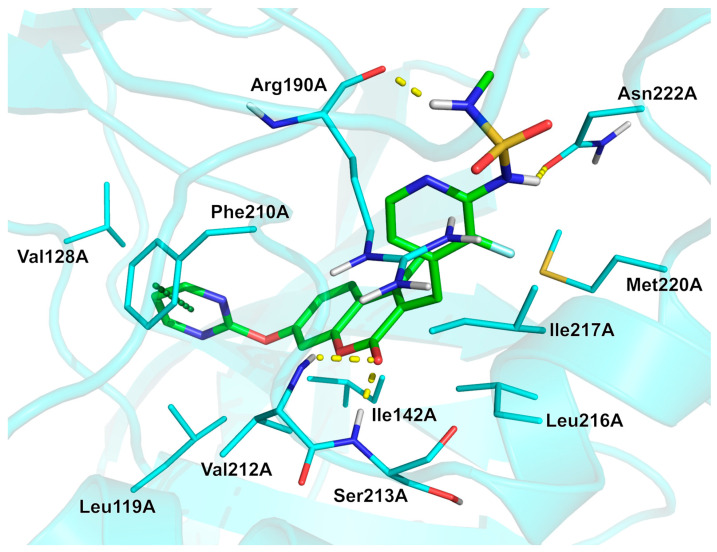
Experimental bound conformation of avutometinib (PDB: 3WIG [51]; human MEK1 kinase in complex with avutometinib and MgAMP-PNP). Avutometinib is depicted as thick green sticks, MEK1 is represented by cyan cartoons and thin sticks. H-bonds are represented by yellow dashed lines, while the dashed dark green line represents a π-π stacking interaction.

Analysis of the experimentally solved MEK1/avutometinib complex (see Figure 5, PDB: 3WIG [51]; human MEK1 kinase in complex with avutometinib and MgAMP-PNP) indicates that the ligand establishes different hydrophobic interactions with residues Leu119A, Val128A, Ile142A, Phe210A, Leu216A, and Ile217A. The bound conformation is stabilized by four H-bonds that involve the sulphonamide NH groups and the backbone carbonyl oxygen of Arg190A, as well as the sidechain oxygen atom of Asn222A, and the lactone carbonyl oxygen and the backbone amide nitrogens of Val212A and Ser213A. In addition, a π-π stacking interaction with the phenyl ring of Phe210A is observed. The fluorine atom may also contribute to stabilization of the bioactive conformation of the ligand by interacting with the sulphonamide NH group and the sulfur and oxygen atoms of Met220A and Asn222A, respectively. Physicochemical parameters of avutometinib (*LogP*: 1.91, tPSA: 130.81, LogS: −5.347) compared to the non-halogenated derivative (*LogP*: 1.77, tPSA: 130.81, LogS: −5.119) showed that the fluorine did not affect the *LogP* value much, but very likely, besides its potential influence on pharmacodynamics, it may act as a metabolic shield, preventing a primary route of degradation.

**Scheme 4:**
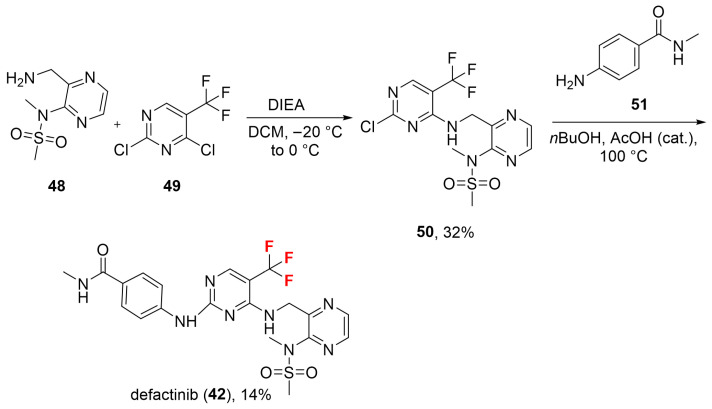
Preparation of defactinib (**42**).

**Figure 6:**
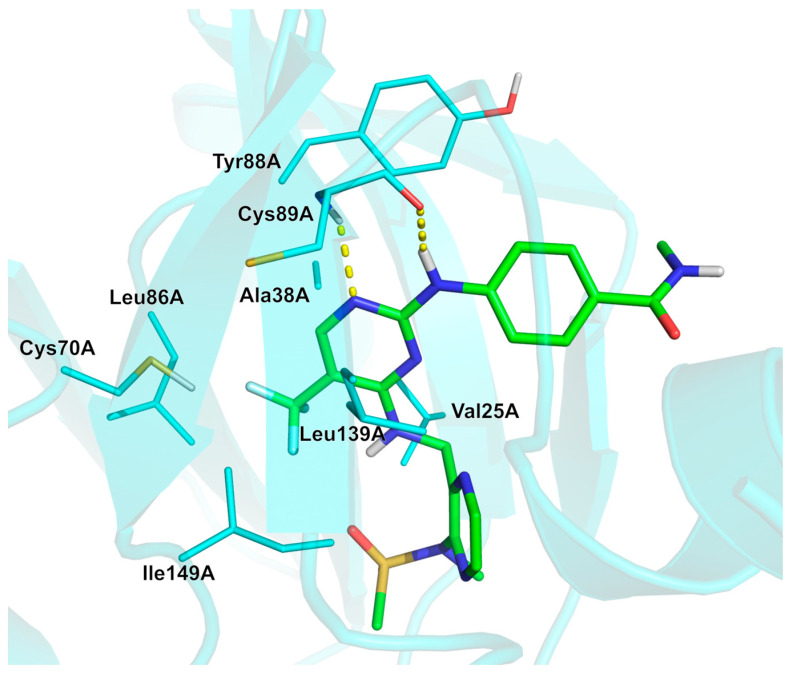
Experimental bound conformation of defactinib (PDB: 5MAH [53]; crystal structure of MELK in complex with defactinib). Defactinib is depicted as thick green sticks, MELK is represented by cyan cartoons and thin sticks. H-bonds are represented by yellow dashed lines.

Defactinib (**42**, Scheme 4) is obtained via reacting *N*-(3-aminomethyl-pyrazin-2-yl)-*N*-methyl-methanesulfonamide **48** with 2,4-dichloro-5-trifluoromethyl-pyrimidine **49** in *N*,*N-*diisopropylethylamine (DIEA)/DCM to afford intermediate **50** in 32% yield. The subsequent S_N_Ar using 4-amino-*N*-methyl-benzamide **51** as a nucleophile in butanol/acetic acid (*n*BuOH/AcOH) afforded the product defactinib (**42**) in 14% yield [52]. In the paper, the aminopyrimidine inhibitor binding was investigated, confirming existing FAK structure–activity relationships and providing a structure–kinetic relationship study for FAK [52]. The –CF_3_ group likely enhances binding affinity with the target protein by improving nonpolar interactions with residues Val25A, Ala38A, Leu86A, and Ile 149A (Figure 6). The three fluorine atoms might even be involved in the stabilization of the bioactive conformation by interacting with one of the ligand sulphone oxygens and with the sidechain sulfur of Cys70A. Furthermore, the pyrimidine ring forms hydrophobic interactions with Ala38A and Leu139A, while the benzene ring forms hydrophobic interactions with Tyr88A. Two H-bonds are observed between the ligand and the backbone of residue Cys89A (Figure 6, PDB: 5MAH [53]; crystal structure of MELK in complex with defactinib). Physicochemical parameters of defactinib (*LogP*: n/a, tPSA: 139.98, LogS: −7.014) compared to non-halogenated derivative (*LogP*: n/a, tPSA: 139.98, LogS: −6.201) showed that the trifluoromethyl group affected solubility but to a small extent.

### 2.4. Taletrectinib (***52***)

**Scheme 5:**
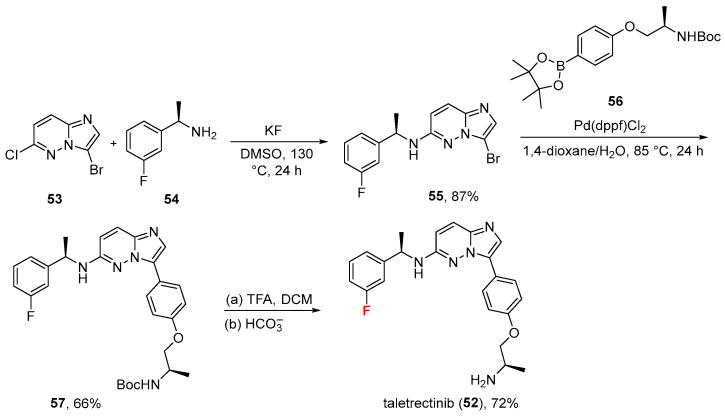
Preparation of taletrectinib (**52**).

Approved on 11 June 2025, taletrectinib (**52**, Ibtrozi^®^, Scheme 5) is a next-generation oral ROS1 and NTRK tyrosine kinase inhibitor (TKI) [54,55]. It was designated to target ROS1 fusion-positive and NTRK fusion-positive non-small cell lung cancer (NSCLC), including brain metastases and resistance mutations [56].

Chemically, it is composed of an imidazo [1,2-b]pyridazine central core decorated by two additional pendants, each containing an aromatic ring. Moreover, an aromatic fluorine atom is present. Its synthesis [57] [taken from its patent assignee and developers: AnHeart Therapeutics (Hangzhou) Co., Ltd. Hangzhou, Zhejiang, China] develops from 3-bromo-6-chloroimidazo [1,2-*b*]pyridazine **53** that is reacted with (R)-1-(3-fluorophenyl)ethan-1-amine **54** in the presence of potassium fluoride (KF) and dimethyl sulfoxide (DMSO) as a solvent to give secondary amine **55** in excellent yield (87%). Then, a Suzuki coupling (promoted by [1,1′-bis(diphenylphosphino)ferrocene]palladium(II) dichloride) between **55** and boronic ester **56** gave BOC-protected amine **57** (66% yield) which was eventually converted to taletrectinib (**52**) via acidic deprotection (trifluoroacetic acid, TFA) followed by neutralization (hydrogen carbonate, HCO_3_^−^) in good yield (72%). The most common treatment-related adverse events were alanine transaminase elevations (72.7%), aspartate transaminase elevations (72.7%), and nausea (50.0%) [55].

In biochemical kinase assays, taletrectinib shows ~20-fold selectivity for ROS1 (IC_50_ = 0.07) over TRKA (IC_50_ = 1.26) and TRKB (IC_50_ =1.47) [58]. Physicochemical parameters of taletrectinib (*LogP*: 3.95, tPSA: 75.24, LogS: −5.467) compared to the non-halogenated derivative (*LogP*: 3.8, tPSA: 75.24, LogS: −5.221) showed that the fluorine has a little impact on lipophilicity and solubility but may exert an essential role for the drug’s potency, selectivity, and pharmacokinetic properties.

### 2.5. Sunvozertinib (***58***)

Sunvozertinib (**58**, Zegfrovy^®^) is an oral, irreversible tyrosine kinase inhibitor (TKI) that selectively targets EGFR exon 20 insertion mutations and certain human epidermal growth factor receptor 2 (HER2) exon 20 insertion mutations, while minimizing activity against wild-type EGFR. It binds covalently to the kinase, disrupting tumor cell proliferation and angiogenesis in non-small cell lung cancer (NSCLC) models [59].

**Scheme 6:**
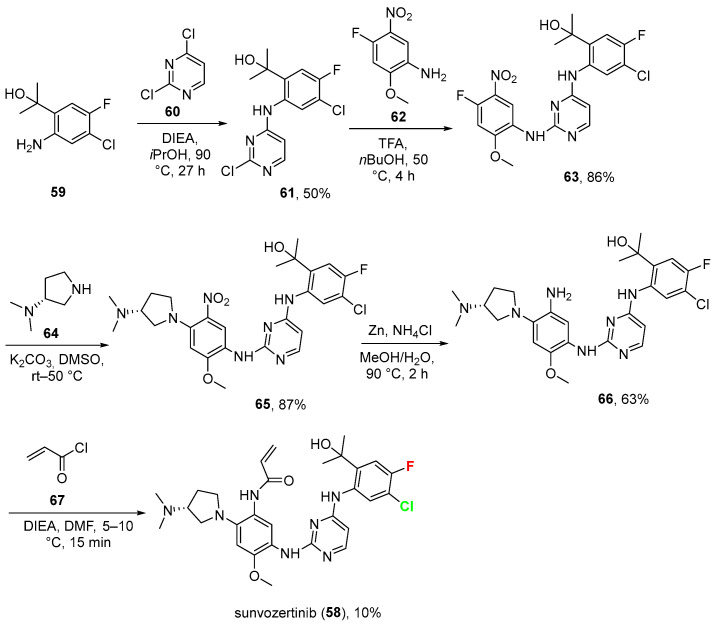
Preparation of sunvozertinib (**58**).

Received FDA accelerated approval on 2 July 2025, under the brand name Zegfrovy^®^, for adult patients with advanced or metastatic NSCLC with EGFR exon 20 insertions, post platinum chemotherapy, as detected by an FDA-approved test [60]. Chemically, it contains one fluorine and one chlorine on the same aromatic ring. Its synthesis [taken from its patent assignee and developers: Dizal (Jiangsu) Pharmaceutical Co., Ltd. Wuxi, Jiangsu Province, China] [61] (Scheme 6) starts from halogenated aniline derivative **59** that is reacted with dichloro-pyrimidine **60** to give trihalogenated intermediate **61** (50% yield), which in turn was treated with 4-fluoro-2-methoxy-5-nitroaniline **62** to give compound **63** in great yield (83%). This was then reacted with (*R*)-N,N-dimethylpyrrolidin-3-amine **64** to yield **65** in excellent yield (87%). The reduction in nitro group operated by metallic zinc (Zn) in an acidic environment (ammonium chloride, NH_4_Cl) gave aniline **66** (63%), which was eventually transformed into Sunvozertinib (**58**) via the addition of acryloyl chloride **67** in the presence of DIEA in a low yield (10%). Common side effects of sunvozertinib include diarrhea, nausea, itchy skin, rash, infected skin around the nail, dry skin, and decreased appetite [62]. Its IC_50_ is 2.1 nM against EGFR exon 20 insertion NPG mutant (in pEGFR phosphorylation assays) [63]. Physicochemical parameters of sunvozertinib (*LogP*: 4.53, tPSA: 113.82, LogS: −7.366) compared to the non-halogenated derivative (*LogP*: 3.81, tPSA: 113.82, LogS: −6.419) showed that the fluorine optimizes the drug’s pharmacokinetic (PK) properties, while the chlorine is a masterstroke of pharmacodynamic (PD) design. The introduction of both 4-fluoro and 5-chloro substitutions successfully reduced human hepatocyte clearance from 34 to 7.1 (μL/min)/(106 cells) [63].

### 2.6. Sebetralstat (***68***)

**Scheme 7:**
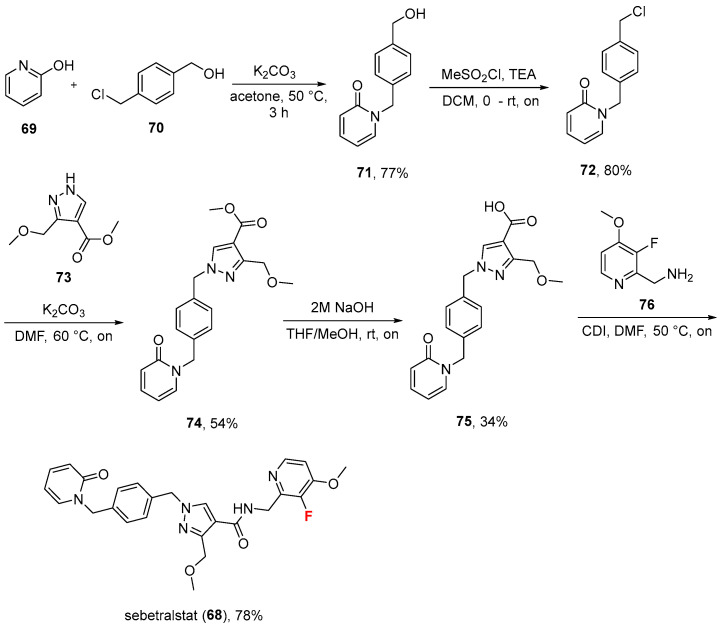
Preparation of sebetralstat (**68**); on = overnight.

Approved on 7 July 2025 by the FDA [64], sebetralstat (**68**, Ekterly^®^, Scheme 7) is an oral drug developed by KalVista Pharmaceuticals (Framingham, MA, USA) for the on-demand treatment of hereditary angioedema (HAE) attacks [64,65]. Hereditary angioedema (HAE) attacks are characterized by sudden, painful swelling episodes in various parts of the body. These attacks can affect the face, limbs, abdomen, and, most seriously, the airways, potentially leading to life-threatening situations [66]. Sebetralstat is a selective plasma kallikrein inhibitor designed to prevent the excessive production of bradykinin [67], a peptide that increases vascular permeability and causes the painful, potentially life-threatening swelling characteristic of HAE. The most common sebetralstat side effect is headache [68].

The synthesis (taken from its patent assignee and developers: KalVista Pharmaceuticals) for sebetralstat [69,70] develops from pyridin-2-ol **69**, which is treated with benzylchloride **70** to give 2-pyridone-based benzyl alcohol derivative **71** in high yield (77%). Conversion of alcohol to chloride derivative **72** via methanesulfonyl chloride (MeSO_2_Cl) (80%) was followed by reaction with pyrazole **73** to afford the desired regioisomer (after column chromatography) **74** (54%). Alkaline hydrolysis of ester using 2M NaOH gave free carboxylic acid **75** (34%), which was eventually coupled with aminomethyl pyridine **76** under CDI conditions to generate sebetralstat (**68**) in good yield (78%). Overall, sebetralstat was associated with encouraging side-effect and safety profiles [71]. Oral sebetralstat provided faster symptom relief, more rapid reduction in severity, and a higher likelihood of complete resolution within 24 h compared to placebo in HAE attacks [71].

**Figure 7:**
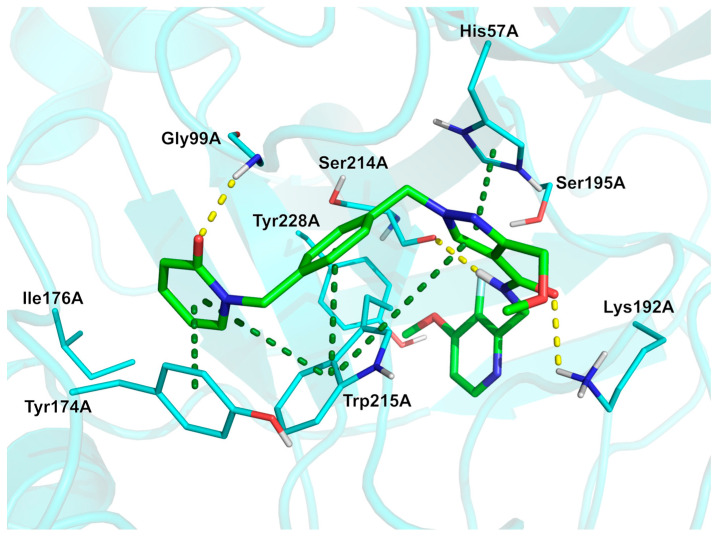
Experimental bound conformation of sebetralstat (PDB: 8A3Q [70]; crystal structure of human plasma kallekrein in complex with sebetralstat). Sebetralstat is depicted as thick green sticks, kallekrein is represented by cyan cartoons and thin sticks. H-bonds are represented by yellow dashed lines, while the dashed dark green lines represent π-π stacking interactions.

Analysis of the experimentally solved kallekrein/sebetralstat complex (Figure 7, PDB: 8A3Q [70]; crystal structure of human plasma kallekrein in complex with sebetralstat) reveals a characteristic U-shaped conformation about the central pyrazole core. This conformation is facilitated by the movement of Trp215A, which enables the formation of a π–π stacking interaction network involving His57A, Tyr174A, Trp215A, and three out of the four ligand aromatic rings. In addition, H-bonds with residues Gly99A, Lys 192A, and Ser214A further stabilize the bound conformation. Fluorine substitution may likely increase metabolic stability and membrane permeability and could also contribute to the stabilization of the bound conformation through interactions with the sidechain oxygen of Ser195A and the backbone carbonyl oxygen of Ser214A. Additionally, Davie et al. [70], based on WaterMap calculations, suggested that the 3-fluoro-4-methoxypyridine might contribute to the favorable binding energy of sebetralstat by displacement of a water molecule they predicted to lie above the face of Tyr228A. The physicochemical parameters of sebetralstat (*LogP*: 2.19, tPSA: 95.83, LogS: −4.422) compared to the non-halogenated derivative (*LogP*: 2.03, tPSA: 95.83, LogS: −4.21) showed that the fluorine has a little impact on these three properties, but may directly contribute to the pharmacokinetic (PK) profile by making the molecule more stable.

### 2.7. Rilzabrutinib (***77***)

**Scheme 8:**
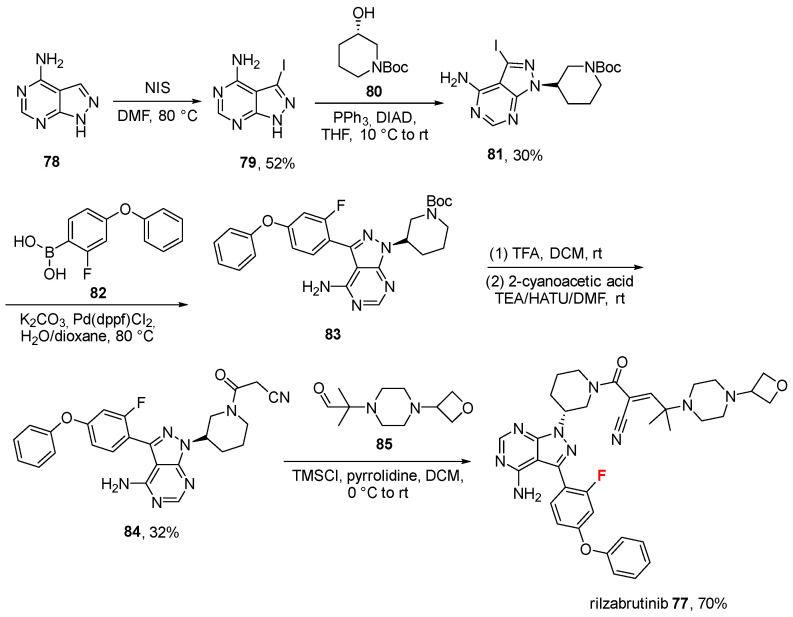
Preparation of rilzabrutinib (**77**).

**Figure 8:**
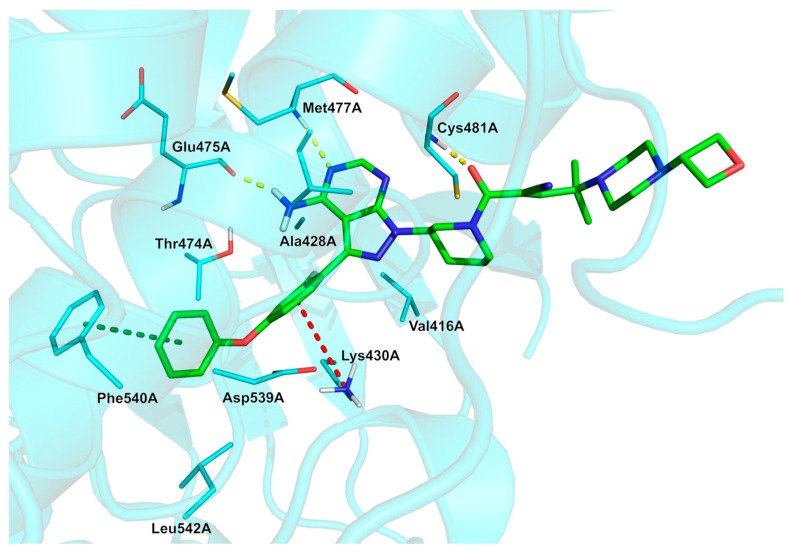
Experimental bound conformation of rilzabrutinib (PDB: 7L5O; crystal structure of the noncovalently bonded complex of rilzabrutinib with BTK). Rilzabrutinib is depicted as thick green sticks, and BTK is represented by cyan cartoons and thin sticks. H-bonds are represented by yellow dashed lines, while π-π stacking and π–cation interactions are represented by dark green and red dashed lines, respectively.

Approved on 29 August 2025, rilzabrutinib (**77**, Wayrilz^®^, Scheme 8) was discovered by Principia Biopharma Inc. (South San Francisco, CA, USA, Sanofi group) for adults with persistent or chronic immune thrombocytopenia (ITP) who have had an insufficient response to a previous treatment [72]. It is an oral active inhibitor of Bruton’s tyrosine kinase BTK (IC_50_ = 1.3 nM), a reversible, covalent, and selective compound [73,74]. It contains an aromatic fluorine atom. Its synthesis (Scheme 8, disclosed by their inventors at Principia Biopharma Inc.) [75] develops from the amine **78**, which is iodinated using *N*-iodosuccinimide (NIS) to obtain intermediate **79** (52% yield). Mitsunobu reaction mediated by triphenylphosphine (PPh_3_)/diisopropyl azodicarboxylate (DIAD) between **79** and *tert*-butyl (*R*)-3-hydroxypiperidine-1-carboxylate (**80**) gave BOC-protected amine **81** (30%). Suzuki coupling reaction of **81** with the boronic acid **82** afforded **83** in good yield (77%), followed by the removal of the BOC protecting group to give. The free piperidine group was condensed with cyanoacetic acid using hexafluorophosphate azabenzotriazole tetramethyl uronium (HATU) and TEA in DMF to give amide **84** (two steps yield: 32%, starting from **83**). Eventually, the crucial step to form α-cyanoacrylamide was a Knoevenagel condensation reaction with the aldehyde **85**, catalyzed by trimethylsilyl chloride (TMSCl), to give the final product rilzabrutinib **77** in good yield (70%). Moving to the SAR of this molecule [76], the pyrazolo [3,4-d]pyrimidine core with the anilino/phenoxy aryl substituent provides the H-bonding to the hinge and main potency. A piperidine linker/amide links the hinge core to the electrophile-bearing side chain and controls vector to Cys481. The warhead represented by the α-cyanoacrylamide moiety, that is, the feature that enables reversible covalent Michael addition to Cys481. Additionally, a tertiary amine/piperazine bearing an oxetane (or similar polar substituent) was identified as the best substituent to tune PK, solubility, and cell penetration. The fluorine atom generates improved human whole blood potency while occupying a small pocket adjacent to Ala428; in general, the complexity of the side chain attached to the pyrimidine ring allows for more selective and reversible binding to the cysteine residue compared to ibrutinib, which has a simpler side chain. The reversible binding of rilzabrutinib leads to enhanced specificity over time since off-target binding is not sustained [77]. The analysis of the rilzabrutinib/BTK complex (Figure 8, PDB: 7L5O; crystal structure of the noncovalently bonded complex of rilzabrutinib with BTK) reveals that the fluorobenzene ring is located adjacent to residues Val416A, Ala428A, Lys430, and Thr474A, forming hydrophobic interactions with Val416A and a π–cation interaction with Lys430A. The terminal benzene ring is found to make hydrophobic interactions with Phe540A and Leu542A, as well as a π–π stacking interaction with Phe540A. The bioactive conformation is stabilized by H-bond interactions with Glu475A, Met477A, and Cys481A. The physicochemical parameters of rilzabrutinib (*LogP*: 3.78, tPSA: 135.38, LogS: −7.724) compared to the non-halogenated derivative (*LogP*: 3.62, tPSA: 135.38, LogS: −7.46) showed that fluorine has little impact on these three properties.

### 2.8. Imlunestrant (***86***)

**Scheme 9:**
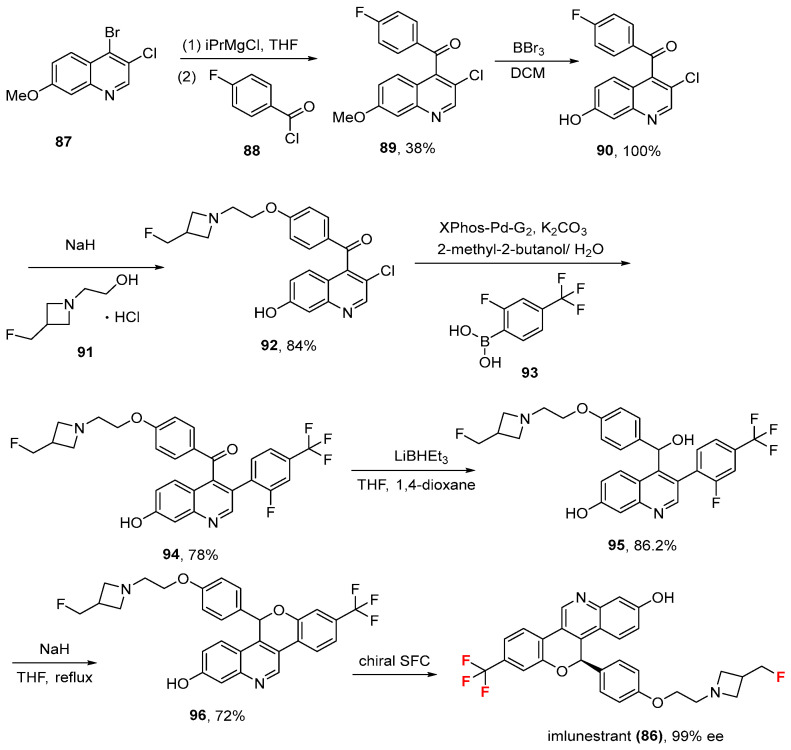
Preparation of imlunestrant (**86**).

Imlunestrant (**86**, Inluriyo^®^, Eli Lilly and Company, Indianapolis, IN, USA), an estrogen receptor antagonist, was approved by the Food and Drug Administration on 25 September 2025 for adults with ER-positive, HER2-negative, ESR1-mutated advanced or metastatic breast cancer who had progressed after at least one line of endocrine therapy [78]. Chemically, it contains an aliphatic fluorine and a trifluoromethyl group bound to an aromatic ring. Its synthesis (Scheme 9, taken from the inventors at Eli Lilly and Company) [79,80] develops from initial metal-halogen exchange of 4-bromo-3-chloro-7-methoxyquinoline **(87**) with isopropylmagnesium chloride (*i*-PrMgCl) in tetrahydrofuran (THF) and subsequent Grignard reaction with 4-fluorobenzoyl chloride (**88**) in THF, which gives (3-chloro-7-methoxy-4-quinolyl)-(4-fluorophenyl)methanone (**89**, 38%). O-demethylation of 7-methoxyquinoline derivative (**89**) by means of Boron tribromide (BBr_3_) in DCM affords 7-hydroxyquinoline derivative (**90**) in quantitative yield, which, upon coupling with 2-[3-(fluoromethyl)azetidin- 1-yl]ethanol hydrochloride (**91**) using sodium hydride (NaH) in DMF, produces the corresponding ether (**92**, 84%). Suzuki coupling of fluoro derivative (**92**) with *ortho*-fluorurated benzeneboronic acid (**93**) by means of catalyst XPhos-Pd-G2 and K_2_CO_3_ in 2-methyl-2-butanol/ H_2_O generates diaryl derivative (**94**, 78%), whose keto moiety is reduced with lithium triethylborohydride (LiBHEt_3_) in THF/1,4-dioxane to produce secondary alcohol (**95**, 86.2%). Intramolecular cyclization of compound (**95**) using NaH in refluxing THF provides racemic chromeno [4,3-c] quinolin-2-ol derivative (**96**, 72%), which is finally subjected to chiral resolution by means of chiral supercritical fluid chromatography (SFC) to give **86** in 99% enantiomeric excess (ee). Imlunestrant induces long-lasting target inhibition and significant arrest of tumor growth [81]. It is important to note that the fluromethylazetidine side chain of imlunestrant may account for its degradation activity on the estrogen receptor [81]. Moreover, their favorable toxicity profiles and oral bioavailability may be particularly relevant for their possible future combination with other targeted treatments, including CDK4/6 inhibitors [81]. The IC_50_ concentrations were 0.34 nM and 5.2 nM in tumor 47, sample D (T47D), and Michigan Cancer Foundation-7 (MCF7) cells, respectively [82]. The physicochemical parameters of imlunestrant (*LogP*: 5.56, tPSA: 54.29, LogS: −7.553) compared to the non-halogenated derivative (*LogP*: 5.4, tPSA: 54.29, LogS: −6.746) showed that the fluorine atoms have a negative impact on solubility, but this is weighted out by an improvement in the binding process and metabolic stability.

### 2.9. Paltusotine (***97***)

The FDA has authorized on 25 September 2025 paltusotine (Palsonify^®^, Crinetics Pharmaceuticals, Inc., San Diego, CA, USA), a new nonpeptide somatostatin receptor type 2 (SST2) agonist, for the treatment of acromegaly in people who had an insufficient response to surgery and/or for whom surgery is not an option [83]. Paltusotine was chosen from a newly synthesized series of compounds due to its potent and highly specific activation of the SST2 receptor, as peptides are not effectively ingested from the gastrointestinal tract [84]. Chemically, it is a diarylquinoline containing two aromatic fluorine atoms.

**Scheme 10:**
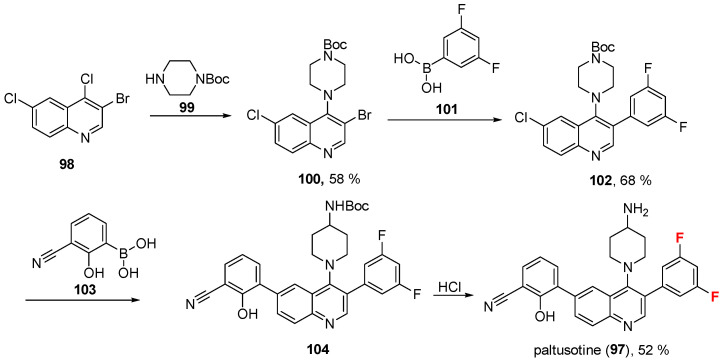
Preparation of paltusotine (**97**).

**Figure 9:**
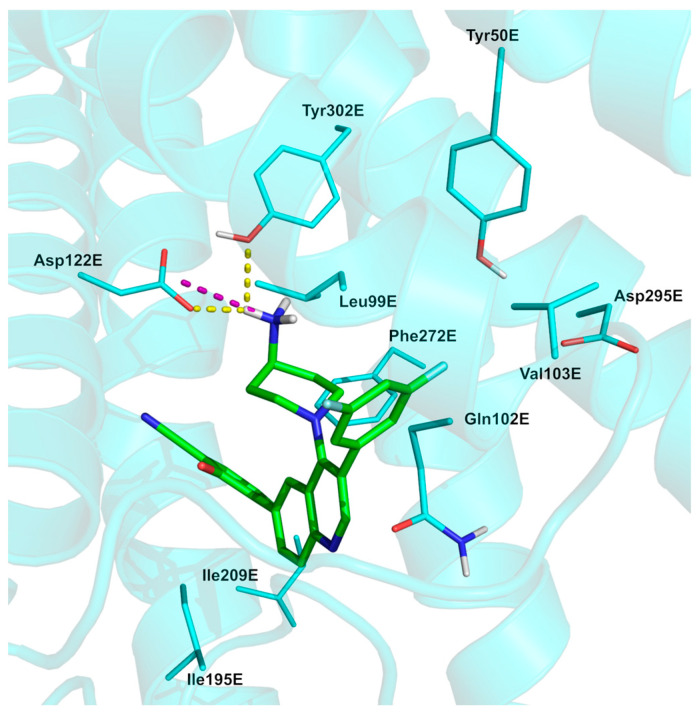
Experimental bound conformation of paltusotine (PDB: 7YAC [85]; CryoEM structure of paltusotine-bound SSTR2-Gi complex). Paltusotine is depicted as thick green sticks, and somatostatin receptor is represented by cyan cartoons and thin sticks. H-bonds are represented by yellow dashed lines, while ionic interaction is represented by purple dashed lines.

Its synthesis [84] (Scheme 10, described by Crinetics Pharmaceuticals) involves four steps to generate the final 4-(4-aminopiperidinyl)-3,6-diarylquinoline. The first step was the nucleophilic replacement on 3-bromo-4,6-dichloroquinoline (**98**) via *N*-BOC-piperazine **99**, which generated tert-butyl (1-(3-bromo-6-chloroquinolin-4-yl)piperidin-4-yl)carbamate **100** (58%). The second step was the selective Suzuki coupling on the bromine via using (3,5-difluorophenyl)boronic acid (**101**) to give synthon **102** (68%). This was subjected to another Suzuki coupling involving (3-cyano-2-hydroxyphenyl)boronic acid **103** to afford *N*-Boc-protected compound **104**, which was eventually converted into paltusotine (**97**, 52%) via acidic treatment (HCl). Although yields were not disclosed, the chemistry suggests they are likely to be high. The analysis of the experimentally solved SSTR2/paltusotine complex (Figure 9, PDB: 7YAC) [85]; CryoEM structure of paltusotine-bound SSTR2-Gi complex) reveals that the primary amino group of aminopiperidin strongly interacts with residue Asp122E, making both a H-bond and a salt bridge, as well as a H-bond with Tyr302E. The 3,5-difluorophenyl moiety of paltusotine is accommodated within a pocket lined by residues Leu99E, Gln102E, and Val103E, establishing hydrophobic contacts with Leu99E and Gln102E. In addition, this moiety engages in Van der Waals interactions with Tyr50E and Asp295E, with the fluorine atom likely interacting with the sidechain oxygen atoms of Tyr50E and Asp295E [86]. As reported by Zhao et al. [85], the latter residue appears to be crucial for ligand activity, as substitution with bulkier groups has been reported to reduce activation potency. Finally, the ligand forms other hydrophobic interactions with Ile195E, Ile209E, and Phe272E.

The SAR studies clearly suggested that a 3,5 difluoro substitution of the benzene ring is preferred for SST2 receptor activity [84]. It is noticeable that deleting both fluoro groups resulted in a seven-fold potency loss [84]. The physicochemical parameters of paltusotine (*LogP*: 4.76, tPSA: 85.64, LogS: −7.627) compared to the non-halogenated derivative (*LogP*: 4.45, tPSA: 85.64, LogS: −7.11) showed that the fluorine atoms have little impact on these properties but are fundamental for the binding.

### 2.10. Remibrutinib (***105***)

Remibrutinib (Rhapsido^®^, Novartis, Basel, Switzerland), an oral, highly selective Bruton’s tyrosine kinase inhibitor, showed efficacy in the treatment of chronic spontaneous urticaria [87]. It was approved by the FDA on 30 September 2025 to treat chronic spontaneous urticaria in adults who remain symptomatic despite H1 antihistamine treatment [88].

Chemical key features include an amino-pyrimidine core linked to a fluorinated phenyl group, a cyclopropyl-fluorobenzamide moiety, and a reactive methylacryloyl group that covalently binds to Bruton’s tyrosine kinase (BTK).

**Scheme 11:**
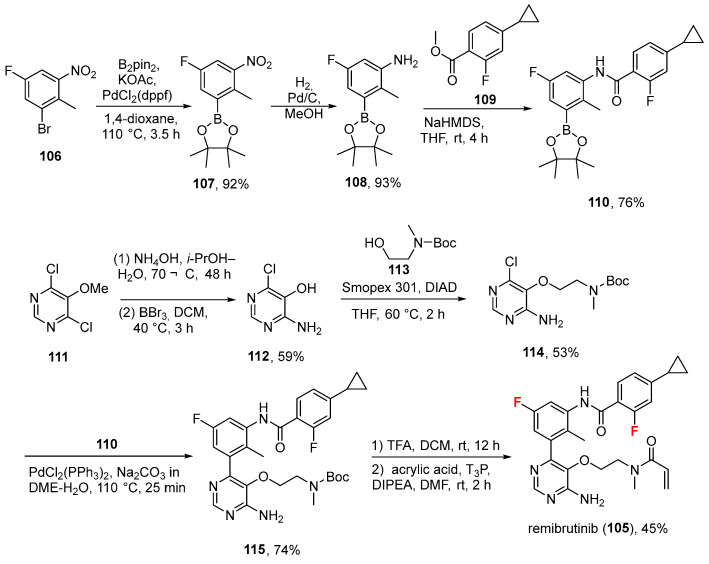
Preparation of remibrutinib (**105**).

Remibrutinib forms a covalent bond with C481 and inhibits BTK with an IC_50_ of 1.3 ± 0.9 nM. Based on affinity measurements (K_d_ = 0.63 nM for BTK), remibrutinib exhibits high selectivity against other similar kinases [89]. Its synthesis [90] (Scheme 11, disclosed by their inventors at Novartis) is a kind of parallel synthesis in which two synthons (compounds **110** and **114**) are prepared in parallel and then joined in the last steps of the synthesis. The first synthon is obtained from 1-bromo-5-fluoro-2-methyl-3-nitrobenzene (**106**), which underwent a Suzuki coupling using bis(pinacolato)diboron (B_2_pin_2_), potassium acetate (KOAc), and PdCl_2_(dppf) in 1,4-dioxane in excellent yield (**107**, 92%). The following reduction (catalytic hydrogenation) of the nitro group afforded aniline **108** (93% yield), which was used to prepare amide **110** by reacting with methyl ester **109** using sodium hexamethyldisilazide (NaHMDS) as the base in THF (76%). The second synthon is obtained via reacting pyrimidine **111** with ammonium hydroxide (NH_4_OH) and BBr_3_ to give monochloro aniline **112** (59%). This was transformed into tertiary amine **114** via a polymer-bound triphenylphosphine (Smopex 310) in the corresponding Mitsunobu reaction involving tert-butyl (2-hydroxyethyl)(methyl)carbamate **113** (53%). This reacted with **110** under Suzuki conditions [(PdCl_2_(PPh_3_)_2_ Na_2_CO_3_ in 1,2-dimethoxyethane/water, DME–H_2_O, 110 °C, 25 min)] to give the precursor **115** for remibrutinib (**105**). The last step involved the deprotection of the BOC group (using TFA) and the formation of acrylamide via propanephosphonic acid anhydride (T_3_P) in a moderate yield (45%). The physicochemical parameters of remibrutinib (*LogP*: 4.28, tPSA: 109.38, LogS: −6.127) compared to the non-halogenated derivative (*LogP*: 3.97, tPSA: 109.38, LogS: −5.607) showed that the fluorine atoms have a slightly negative impact on solubility and increase lipophilicity, and may be fundamental as an aromatic metabolic blocker.

### 2.11. Nerandomilast (***116***)

On 7 October 2025, the FDA approved nerandomilast (Jascayd^®^, Boehringer Ingelheim, Ingelheim, Germany) for the treatment of adults with idiopathic pulmonary fibrosis [91].

Nerandomilast (also known as BI 1015550) is an orally administered, preferential inhibitor of phosphodiesterase 4B with antifibrotic and immunomodulatory properties. It has been proven that **116** decreases the course of idiopathic pulmonary fibrosis, but further investigation into its effects in other kinds of progressive pulmonary fibrosis is required [92].

The synthesis of nerandomilast (disclosed by their inventors at Boehringer Ingelheim) is a complex process designed to be robust and scalable for multikilogram production. The strategy involves assembling three key components: a dihydrothieno [3, 2-d]pyrimidine core, an aminocyclobutanol moiety, and a 5-chloro-2-(piperidin-4-yl)pyrimidine group [93].

**Scheme 12:**
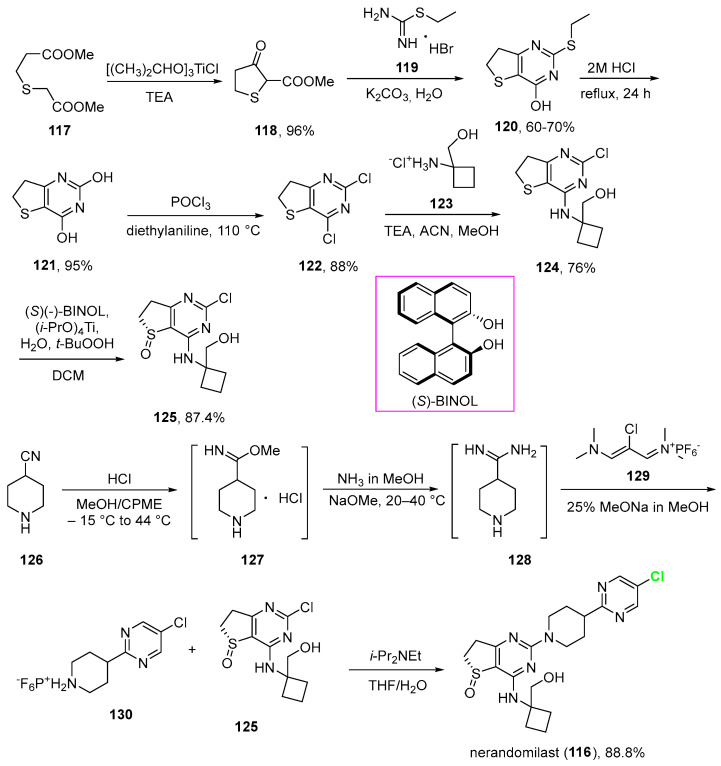
Synthesis of nerandomilast (**116**).

The preparation of dihydrothieno [3, 2-d]pyrimidine core **122** (Scheme 12) starts with thioether **117** that was treated with Lewis acid chlorotriisopropoxytitanium [(CH_3_)_2_CHO]_3_TiCl] and triethylamine to give **118** in an improved intramolecular Dieckmann with excellent yield (96%). This was reacted with the relatively expensive ethylisothiourea hydrobromide **119** to afford pyrimidine **120** in good yield (60–70%). This was followed by acidic hydrolysis to give diol **121** in excellent yield (95%) [94]. The authors pointed out that there was significant decomposition of **120** under the basic reaction conditions that resulted in the formation of foul-smelling byproducts. Diol **121** was eventually converted to dichloro-pyrimidine **122** by reacting with phosphorus oxychloride (POCl_3_) (88%).

The installation of the aminocyclobutanol moiety on dihydrothieno [3, 2-d]pyrimidine core (Scheme 12) was accomplished via reacting **122** with commercially available 1-aminocyclobanemethanol hydrochloride (**123**) to give aminocyclobutanol **124** (76%). The subsequent asymmetric oxidation to chiral sulfoxide was carried out using chiral [1,1′-Binaphthalene]-2,2′-diol, (S)-BINOL, (*i*-PrO)_4_Ti, and with *t*-BuOOH as the stoichiometric oxidant, providing sulfoxide **125** in good yield (87.4%) and enantioselectivity (>99.0% ee). The author added that it is likely the presence of the secondary amine near the sulfide in **124** likely contributed to the strong enantioselectivity obtained.

For the preparation of the 5-chloro-2-(piperidin-4-yl)pyrimidine group (Scheme 12), the synthesis was developed from commercially available piperidine-4-carbonitrile **126**. This was treated with HCl and methanol/cyclopentyl methyl ether (CPME) to give imidate hydrochloride **127**, which, without isolation, was treated with sodium methoxide and ammonia in methanol to give amidine **128**. This was not isolated but condensed with vinamidinium salt **129** in the presence of additional sodium methoxide to give crude **130** as the hexafluorophosphate salt **130**.

Robust and scalable processes were developed for the synthesis of intermediates **125** and **130**, which were transformed into nerandomilast (**116**) in the presence of Hünig’s base and a solvent system consisting of a mixture of THF and water (Scheme 12). An additional recrystallization from 2-propanol/water was required to obtain nerandomilast with the desired single-crystalline polymorph.

The physicochemical parameters of nerandomilast (*LogP*: 1.85, tPSA: 102.01, LogS: −3.99) compared to the non-halogenated derivative (*LogP*: 1.3, tPSA: 102.01, LogS: −3.336) showed that the chlorine atom negatively impacts the lipophilicity and the solubility, but may be fundamental as a steric anchor to achieve the potency and for improving metabolic stability.

### 2.12. Elinzanetant (***131***)

**Figure 10:**
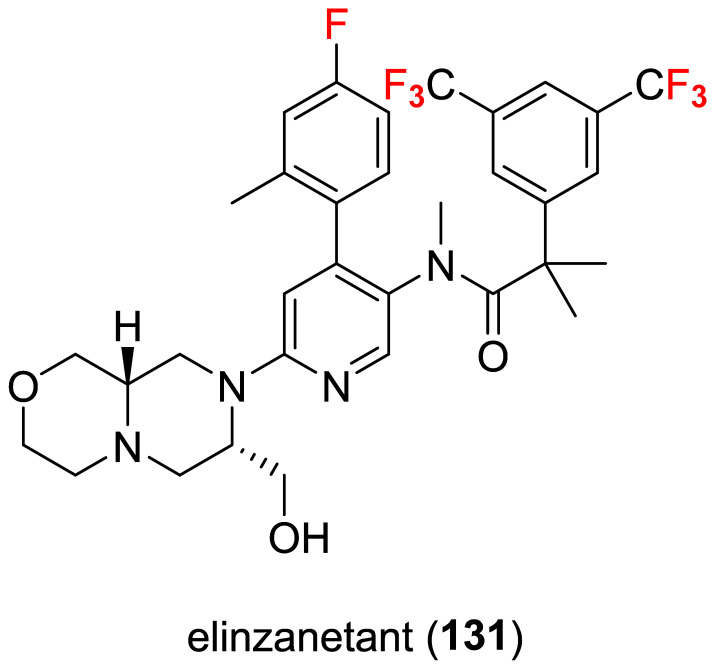
The chemical structure of elinzanetant (**131**).

On 24 October 2025, the FDA approved elinzanetant (Figure 10, Lynkuet^®^, Bayer, Leverkusen, Germany) for the treatment of women with moderate-to-severe vasomotor symptoms due to menopause (IPF) [95].

Elinzanetant was discovered through Bayer’s internal neurokinin receptor antagonist program, originally targeting central nervous system pathways involved in thermoregulation and hormone feedback. Optimization efforts focused on developing a compound that could simultaneously block neurokinin-1 (NK1) and neurokinin-3 (NK3) receptors [96,97]—both implicated in menopausal vasomotor symptoms (VMS) such as hot flashes—while maintaining favorable pharmacokinetics and safety [98]. The compound was selected for development after promising preclinical results showing potent inhibition of substance P [99] and neurokinin B signaling pathways, with good oral bioavailability and central nervous system penetration [100].

Chemically, it displays seven fluorine atoms (two trifluoromethyl groups and an aromatic fluorine).

The patent EP4431512A1 [101] mentions “methods of preparing said compounds, intermediate compounds useful for preparing said compounds”, but is somewhat general and is directed to a family of compounds (not exclusively elinzanetant). Therefore, the specific step-by-step synthesis for the marketed molecule may be partly confidential.

**Scheme 13:**
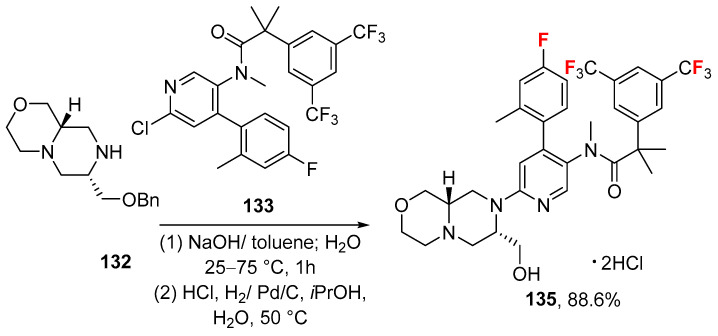
The last steps of synthesis for elinzanetant (**131**). The hydrochloride salt of elinzanetant (**135**).

However, the last steps of its synthesis [102] (Scheme 13, current patent assignee: Kandy Therapeutics) stem from the complex octahydropyrazino [2,1-c][1,4]oxazine heterocycle **132** that is mixed with the chloro-derivate **133** to give protected alcohol **134** in good yield (88.2%), which is eventually converted to elinzanetant hydrochloride salt (**135**) in excellent yield (88.6%) via hydrogenation and formation of salt using HCl. The physicochemical parameters of elinzanetant (*LogP*: 6.92, tPSA: 68.61, LogS: −8.461) compared to the non-halogenated derivative (*LogP*: 5.89, tPSA: 68.61, LogS: −6.867) showed that the fluorine atoms strikingly affect lipophilicity and solubility, but may directly contribute to the pharmacodynamic (PD) and pharmacokinetic (PK) profiles.

### 2.13. Ziftomenib (***136***)

**Figure 11:**
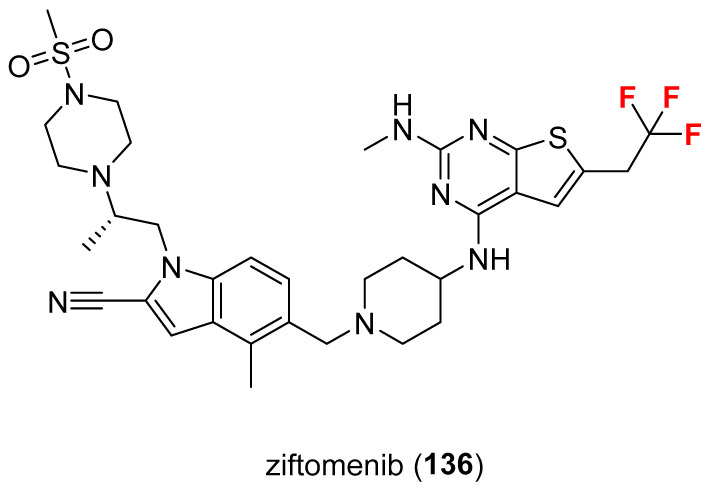
The chemical structure of ziftomenib (**136**).

On 11 November 2025, the FDA approved ziftomenib (Figure 11, Komzifti^®^, Kura Oncology, San Diego, CA, USA and Kyowa Kirin, Tokyo, Japan) for the treatment of adults with relapsed or refractory acute myeloid leukemia with a susceptible nucleophosmin 1 mutation who have no satisfactory alternative treatment options [103]. Ziftomenib selectively disrupts the menin–lysine methyltransferase 2A (KMT2A) interaction [104], which is required for the maintenance of oncogenic transcriptional programs driving leukemic cell proliferation. Ziftomenib shows high selectivity and potency for menin–KMT2A binding, exhibits excellent oral bioavailability, and once-daily pharmacokinetics demonstrates robust differentiation activity without excessive cytotoxicity [105].

Ziftomenib is manufactured via a multi-step chemical process, although the specifics are private. Kura Oncology, the creator of this experimental medication, is responsible for its manufacture. Chemically, it contains a trifluoromethyl pendant which is linked to thieno [3-d]pyrimidine core via a methylene group. Research publications discuss the synthesis of similar compounds, such as thieno [3-d]pyrimidines [106], as part of the development process for menin inhibitors like ziftomenib.

**Scheme 14:**
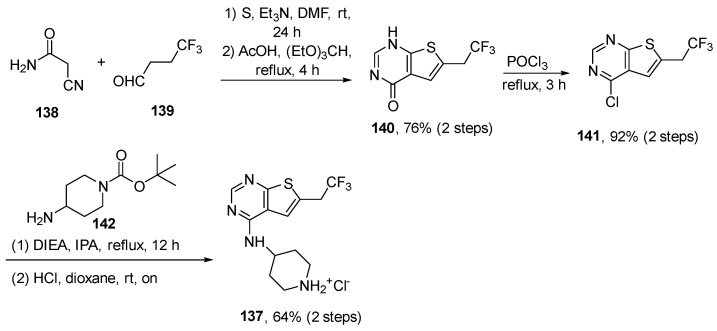
The steps for the synthesis of synthon **137**.

**Figure 12:**
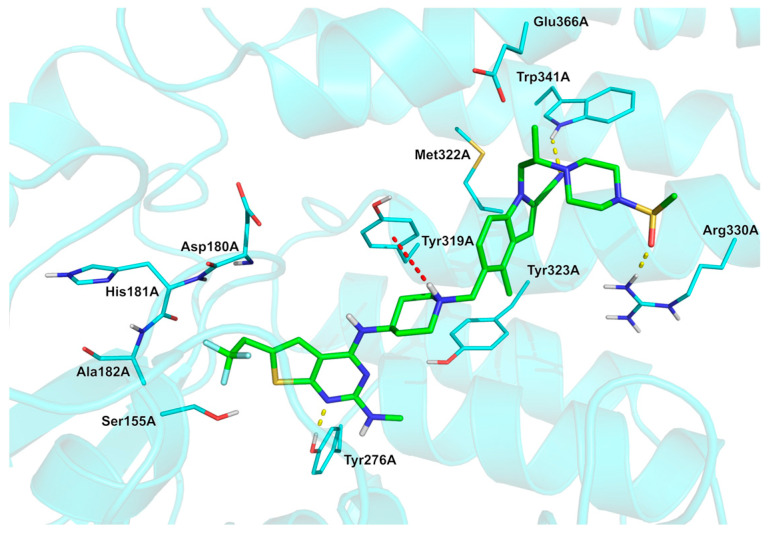
Experimental bound conformation of ziftomenib (PDB: 8VA6 [104]; crystal structure of menin in complex with ziftomenib (KO-539)). Ziftomenib is depicted as thick green sticks, menin is represented by cyan cartoons and thin sticks. H-bonds are represented by yellow dashed lines, while red dashed lines represent π–cation interaction.

The common synthon **137** (Scheme 14), used in the synthesis of various thieno [3-d]pyrimidines [106] was prepared in five steps starting from a Gewald thiophene reaction involving 2-cyanoacetamide **138** and 4,4,4-trifluorobutanal **139** using sulfur, triethylamine, and DMF as a solvent. Condensation of thiophene with triethyl orthoformate gave pyrimidinone **140** (76%, two steps). The conversion of **140** to chloropyrimidine **141** was mediated by POCl_3_ with an excellent yield (92%). The following transformation of **141** to synthon (hydrochloride salt) **137** was accomplished via firstly reacting with tert-butyl 4-aminopiperidine-1-carboxylate **142** and secondly via deprotecting the BOC-group under acidic conditions (HCl). Yield for synthon 137 was 62% (two steps). The analysis of the experimental solved ziftomenib/menin complex (Figure 12, PDB: 8VA6 [104]; crystal structure of menin in complex with ziftomenib) reveals that the ligand establishes hydrophobic interactions with Tyr319A, Met322A, Tyr323A, and Glu366A. In addition, three H-bonds are identified with Tyr276A, Arg330A, and Trp341A, along with a π–cation interaction between the protonated nitrogen atom of piperidine and the phenyl ring of Tyr319A. The –CF_3_ group appears to be accommodated within a pocket lined by residues Ser155A, Asp180A, His181A, and Ala182A. The three fluorine atoms may also contribute to the stabilization of the ligand–menin complex through interactions with the sidechain oxygen of Ser155A, the backbone carbonyl oxygen of Asp180A, the backbone carbonyl oxygen and amide nitrogen of His181A, and the backbone amide nitrogen of Ala182A. The physicochemical parameters of ziftomenib (*LogP*: n/a, tPSA: 119.67, LogS: −8.434) compared to the non-halogenated derivative (*LogP*: 3.86, tPSA: 119.67, LogS: −7.51) showed that the trifluoromethyl group decreases the water solubility but is fundamental for enhancing the binding with KMT2A.

### 2.14. Sevabertinib (***143***)

**Scheme 15:**
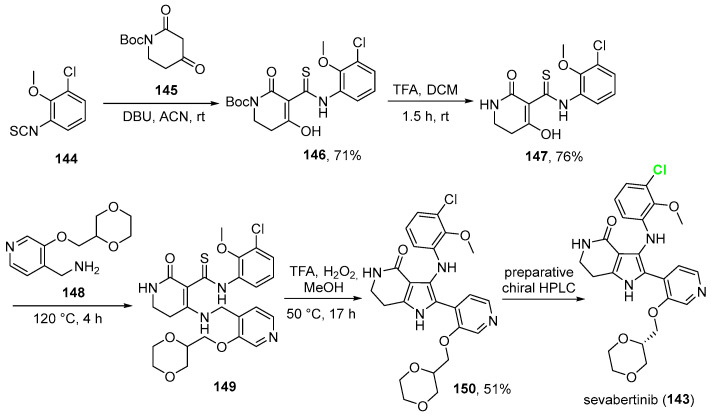
The steps for the synthesis of sevabertinib (**143**).

On 19 November 2025, the FDA approved sevabertinib (Scheme 15, Hyrnuo^®^, Bayer HealthCare Pharmaceuticals Inc.), a kinase inhibitor for patients with metastatic or locally advanced non-squamous non-small cell lung cancer (NSCLC) whose tumors include activating mutations in the HER2 (ERBB2) tyrosine kinase domain (TKD) [107]. Clinical research demonstrating sevabertinib’s ability to reduce tumor size and manage illness supports the FDA’s ruling. Over half of the patients in these studies saw tumor shrinkage, and in one research group, the response rate exceeded 70%. Approximately 80% of the patients experienced disease stabilization or reduction [108]. Diarrhea was the most common adverse event [109]. Chemically, it is a 4H-pyrrolo[3,2-c]pyridin-4-one, which displays a substituted chlorobenzene. Its synthesis [110] (Scheme 15, current patent assignee: Bayer Pharmaceuticals Inc.) develops from 1-chloro-3-isothiocyanato-2-methoxybenzene **144**, which was reacted with tert-butyl 2,4-dioxopiperidine-1-carboxylate **145** in acetonitrile and 1,8-diazabicyclo[5.4.0]undec-7-ene (DBU) as a base to afford oxo-dihydropyridine **146** in 71% yield. Deprotection of BOC via TFA in DCM gave intermediate **147** in 78% yield. This was reacted with 1-{3-[(1,4-dioxan-2- yl)methoxy]pyridin-4-yl}methanamine (**148**) at elevated temperature (120 °C) to yield precursor **149** in 39% yield. The final compound in a racemic mixture (**150**) was obtained via cyclization of **149** in TFA/H_2_O_2_ in methanol (51% yield). Sevabertinib was eventually obtained by a preparative chiral HPLC method.

The physicochemical parameters of sevabertinib (*LogP*: 1.11, tPSA: 102.44, LogS: −5.21) compared to its non-halogenated derivative (*LogP*: 0.56, tPSA: 102.44, LogS: −4.524) showed that the chlorine impacts the lipophilicity (making the molecule more lipophilic) and the solubility (decreases the solubility). However, it is outstanding to see how the *LogP* for this derivative is quite low compared to other described drugs. This is due to a combination of polar groups present in the molecule: dioxane pendant, pyridine nitrogen, and 6-oxo-3,6-dihydropyridine core.

### 2.15. Zoliflodacin (***151***)

**Scheme 16:**
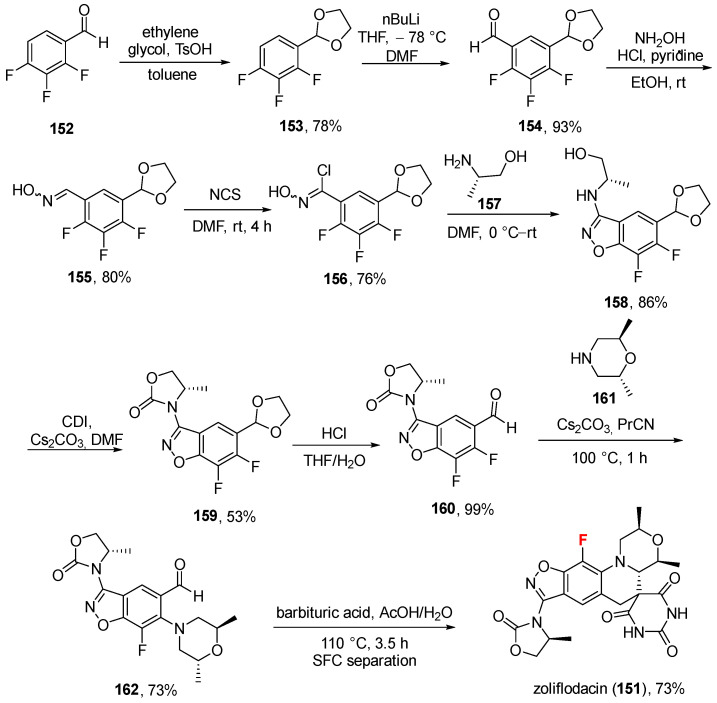
The steps for the synthesis of zoliflodacin (**151**).

On 12 December 2025, the FDA approved zoliflodacin (Scheme 16, Nuzolvence^®^, AstraZeneca, Cambridge, UK) to treat uncomplicated urogenital gonorrhea due to Neisseria gonorrhoeae [111,112]. It is a first-in-class oral antibiotic developed for the treatment of uncomplicated gonorrhea, including infections caused by strains resistant to existing antibiotics [113]. It belongs to a novel chemical class known as spiropyrimidinetriones and works by inhibiting the bacterial DNA gyrase (specifically the GyrB subunit), a mechanism that is distinct from fluoroquinolones and helps avoid cross-resistance [114,115]. The drug is intended to be used as a single-dose oral therapy, which improves patient adherence and removes the need for injectable treatment [111]. Zoliflodacin was originally discovered at AstraZeneca and later advanced through clinical development by Entasis Therapeutics. Its final development and path toward regulatory approval were supported through a public–private partnership between the Global Antibiotic Research and Development Partnership (GARDP) and Innoviva Specialty Therapeutics [116]. Its synthesis [117] (Scheme 16, current patent assignee: AstraZeneca) develops from 2,3,4-trifluorobenzaldehyde **152**, which is treated with ethylene glycol in the presence of catalytic *p*-toluenesulfonic acid (TsOH), giving the 1,3-dioxolane **153** (78%). This was metalated by *n*-butyllithium in cold THF, followed by the addition of DMF to generate aldehyde **154** (93%). This was treated with hydroxylamine hydrochloride to give oxime **155** in high yield (80%), followed by chlorination with *N*-chlorosuccinimide (NCS) to give **156** as a white solid (76%). The treatment of **156** with (*S*)-2-aminopropan-1-ol **157** in DMF afforded **158** via addition to the electrophilic oxime carbon and simultaneously an intramolecular S_N_Ar (oxygen from aminopropan-1-ol displaced one aromatic fluorine). Next, the treatment with CDI and Cs_2_CO_3_ in DMF afforded the characteristic oxazolidinone **159** in moderate yield (53%). Next, the deprotection of dioxolane via HCl in THF/water regenerated aldehyde **160** (quantitative yield), which was treated with (2*R*,6*R*)-2,6-dimethylmorpholine **161** in alkaline conditions (Cs_2_CO_3_) to give the substituted fluoroarene **162** in good yield (73%). Eventually, the addition of barbituric acid to **162** involved a 1,5-hydrogen shift to generate an intermediate that epimerized before cyclization to the spiropyrimidinedione in zoliflodacin **151**. It is worth noting that the last step involved supercritical fluid chromatography (SFC) as a purification technique.

**Figure 13:**
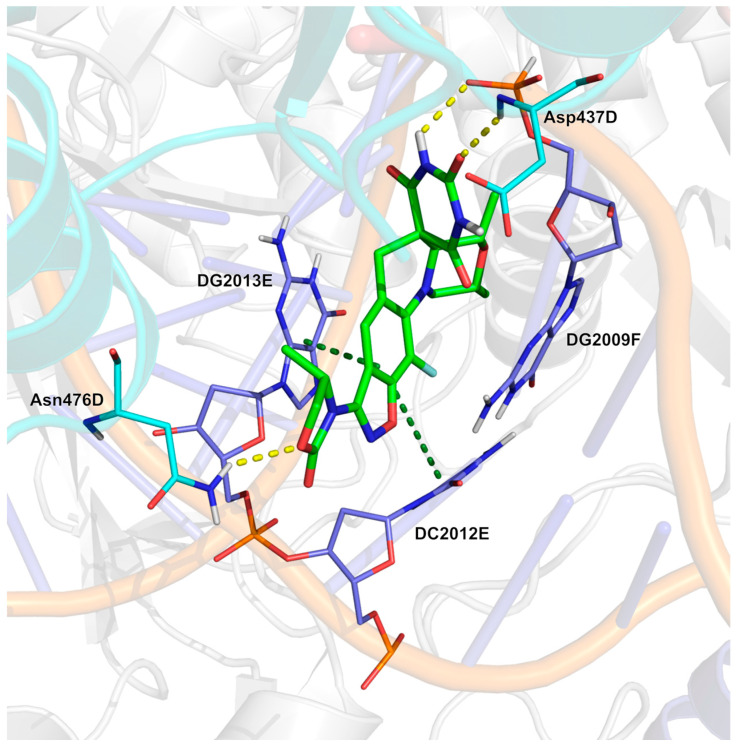
Experimental bound conformation of zoliflodacin (PDB: 8BP2 [118]; crystal structure of zoliflodacin with *S. aureus* DNA gyrase and DNA). Zoliflodacin is depicted as thick green sticks, GyrA subunit is represented by gray cartoons, GyrB by cyan cartoons and thin sticks, substrate DNA by orange cartoons and purple sticks. H-bonds are represented by yellow dashed lines, while the dashed dark green lines represent π-π stacking interactions.

Analysis of the crystal structure of zoliflodacin in complex with *S. aureus* DNA gyrase and substrate DNA (Figure 13, PDB: 8BP2 [118]; crystal structure of zoliflodacin with *S. aureus* DNA gyrase and DNA) reveals that the benzoxazol ring of the ligand engages in π-π stacking interactions with substrate DNA bases DC2012E and DG2013E. Three H-bonds are observed with residues Asp437D, Asn476D, and the phosphodiester backbone of DG2009F.

The physicochemical parameters of zoliflodacin (*LogP*: 1.14, tPSA: 138.87, LogS: −4.792) compared to its non-halogenated derivative (*LogP*: 0.98, tPSA: 138.87, LogS: −4.571) showed that the fluorine impacts the lipophilicity (making the molecule more lipophilic) and the solubility (decreases the solubility).

### 2.16. Tradipitant (***163***)

**Scheme 17:**
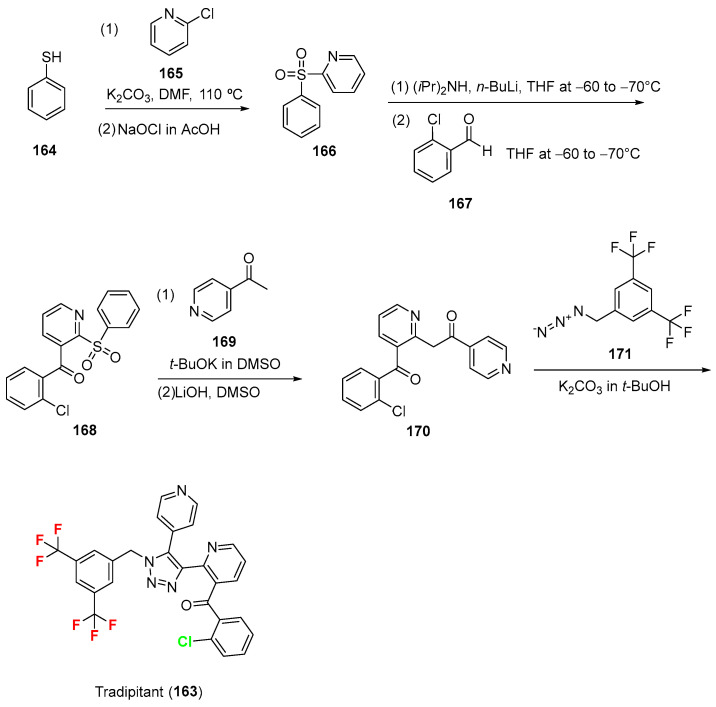
The steps for the synthesis of tradipitant (**163**).

Tradipitant (Scheme 17, NEREUS^®^, developed by Eli Lilly and Company) is a neurokinin-1 (NK-1) receptor antagonist primarily used for the acute prevention of vomiting induced by motion [119]. Approved on 30 December 2025, it works by selectively blocking neurokinin-1 (NK-1) receptors in the central nervous system, which are critical in the sensory pathways that trigger nausea and vomiting [120]. Vanda Pharmaceuticals is the company that licensed it and is developing and commercializing it today [121]. Chemically, it contains two trifluoromethyl groups and a chloroarene. Its synthesis [122] develops from thiophenol **164**, which is condensed with **165** in the presence of K_2_CO_3_ in DMF at 110 °C to yield a sulfide intermediate, which is then oxidized by means of NaOCl in AcOH to give 2-(benzenesulfonyl)pyridine **166**. This is treated with (*i*Pr)_2_NH and *n*-BuLi in THF at −60 to −70 °C and subsequently couples with 2-chlorobenzaldehyde **167** in THF at −60 to −70 °C to furnish (2-(phenylsulfonyl)pyridin-3-yl)-(2-chlorophenyl)methanone **168**. Ketone couples with the enolate of 4-acetylpyridine **169** (formed by treating 4-acetylpyridine with *t*-BuOK in DMSO) in the presence of LiOH in DMSO and subsequently is treated with PhCOOH in iPrOAc to give rise to pyridine benzoate derivative **170**. This finally couples with 1-azidomethyl-3,5-bistrifluoromethylbenzene **171** in the presence of K_2_CO_3_ in *t*-BuOH to afford the title compound tradipitant **163**. The yields for this pathway were not retrieved. The physicochemical parameters of tradipitant (*LogP*: 6.9, tPSA: 69.75, LogS: −9.479) compared to its non-halogenated derivative (*LogP*: 5.47, tPSA: 69.75, LogS: −7.536) indicate that tradipitant exhibits higher lipophilicity and lower aqueous solubility, while both compounds possess identical polar surface area.

## 3. Other Halogen-Containing Approved Drugs in 2025 (Macromolecules)

Datopotamab deruxtecan (dato–DXd; datopotamab deruxtecan–dlnk) is an antibody–drug conjugate (ADC) designed for the targeted treatment of various cancers, particularly non-small cell lung cancer (NSCLC) and triple-negative breast cancer (TNBC) [123,124].

**Figure 14:**
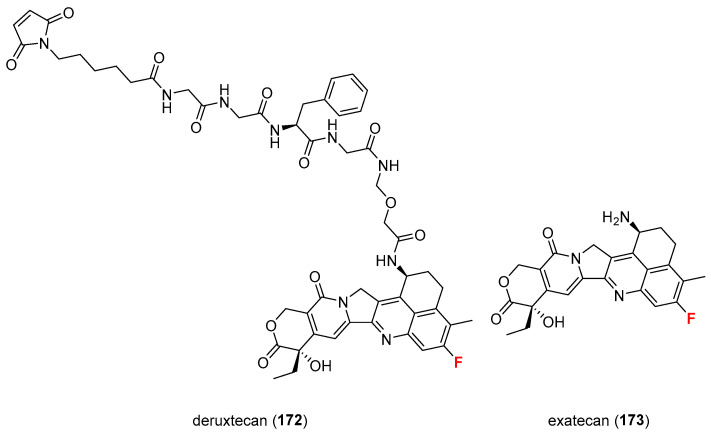
Chemical structure of deruxtecan and exatecan.

Deruxtecan (**172**) is a chemical compound, a camptothecin-derived drug, specifically a derivative of exatecan **173** (Figure 14), that acts as a topoisomerase I inhibitor [123]. It contains an aromatic fluorine, and its molecular weight is 1034.068 g·mol^−1^. The introduction of fluorine into such compounds is likely to improve their pharmacokinetic and pharmacological properties, enhancing antitumor activity.

## 4. Other Halogen-Containing Pipeline Drugs (Not Approved)

**Figure 15:**
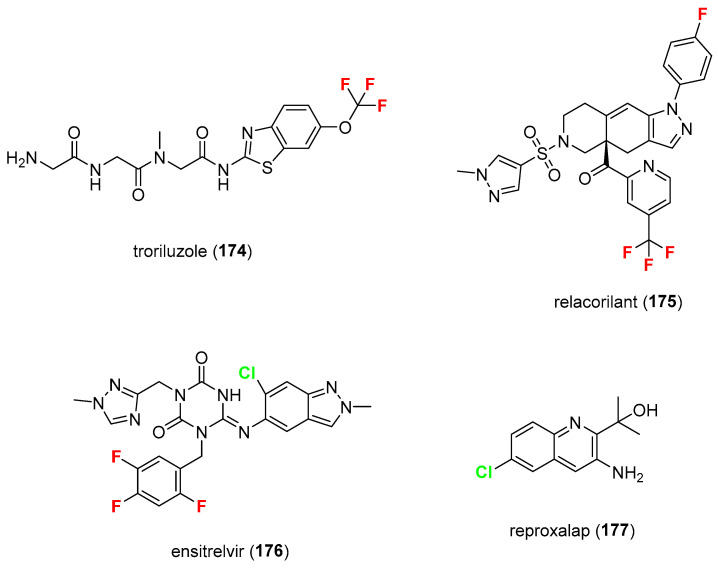
Chemical structure of other halogen-containing molecules in the pipeline.

In this section, a short list of halogen-containing drugs that were not approved but are evaluated for possible approval is presented (Figure 15). Troriluzole **174** is a glutamate modulator in development for the treatment of adult patients with spinocerebellar ataxia [125]. Relacorilant **175** is a selective cortisol modulator under development for use in combination therapy for patients with platinum-resistant ovarian cancer with endogenous hypercortisolism (Cushing’s syndrome) [126]. Ensitrelvir **176** is a 3CL protease inhibitor antiviral in development for post-exposure prophylaxis of COVID-19 [127]. Reproxalap **177** is a novel small-molecule regulator of reactive aldehyde species (RASP) that is being developed to treat dry eye complaints [128]. Troriluzole and relacorilant are fluorine-based small molecules (both contain a trifluoromethyl group), whereas reproxalap is chlorine-based. Ensitrelvir contains both chlorine and fluorine atoms. All four are drug candidates in clinical development or regulatory review and are not currently approved (in the U.S. or broadly globally) for their intended uses.

Small molecules continue to be at the center of the research, according to the FDA’s Novel Drug Approvals for 2025 report. Among 46 total novel drug approvals (data assessed on 30 December 2025), 31 are small molecules (<1000 Da) (67%). It is an impressive reminder that while new modalities grab headlines, organic chemistry continues to drive the majority of therapeutic breakthroughs.

**Figure 16:**
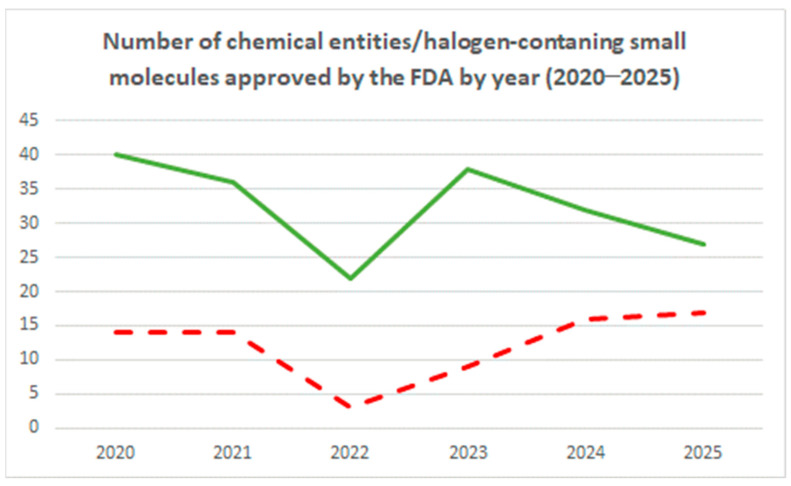
New FDA chemical entities (green line) and halogen-containing small molecules (red dashed line) approved in the years 2020–2025.

An analysis of drugs (new chemical entities) approved by the FDA [129] in the last 6 years are shown in Figure 16. It is evident from the graph that in 2022, this represented a major drop, followed by a recovery in the period 2023–2024 [130]. This trend is also followed by the number of halogen-containing small molecules, which are 16, 14, 6, 14, and 16 for the years 2020, 2021, 2022, 2023, and 2024, respectively [5]. Halogen-containing molecules, within the context of all FDA-approved chemical entities, represent a significant proportion (~40%) in 2020 and 2021, reducing to 27% in 2022 and increasing to a value of ~40% in 2023 and of 50% in 2024. In 2025, among 27 new chemical entities (excluding peptides, oligonucleotides, and antibody–drug conjugates), 17 were halogen-containing small molecules. This represents almost 63%, indicating that halogens have been extensively employed in drugs in the last year. All the small molecules presented in this review (Table 1) are administered orally. Oral medications can be self-administered, reducing the need for clinical supervision and lowering healthcare costs.

About half of the discussed drugs (mirdametinib, avutometinib, defactinib, taletrectinib, sunvozertinib, and imlunestrant) are primarily anticancer drugs, while the rest target autoimmune, endocrine, or other non-cancer conditions.

In this review, we showed that over the decades, halogen atoms have established themselves as silent architects of molecular design, being subtle yet profoundly influential in shaping the behavior of countless therapeutic agents. As of 2025, the continued prevalence of halogen-containing drugs among FDA-approved small molecules highlights how indispensable these elements remain to medicinal chemistry, even in the field of orphan drugs, as already shown by our previous review [131]. Their ability to modulate electronic properties, fine-tune lipophilicity, and guide molecular recognition within biological targets makes them far more than mere substituents; they are deliberate design elements that bridge chemistry and biology.

Recent approvals illustrate an evolving sophistication in halogen employment. Fluorine, in particular, continues to dominate as a tool for enhancing metabolic stability and optimizing pharmacokinetics, while chlorine and iodine often play crucial roles in achieving target selectivity or favorable binding orientations. Bromine was not encountered in any of the approved drugs in 2025. The strategic placement of halogens has thus become a defining feature in modern drug design, supporting the creation of highly optimized therapeutic profiles across oncology, infectious disease, metabolic disorders, and neurological conditions. Moreover, besides the high recurrence of halogens in drug design, a very recent machine-learning study [132] (using a model called Hybrid Dynamic Graph-based Ensemble Model, HD-GEM) actually challenged the old assumption that all halogenation increases toxicity. The findings, particularly regarding complex aromatic scaffolds, suggest that polyhalogenation (adding multiple halogens) can show reduced toxicity, likely due to the stabilizing effect mentioned above, which mitigates the formation of reactive metabolites. Iodine substituents, in particular, were found to be associated with the lowest toxicity in the scaffolds they studied. Additionally, several studies suggest that halogenation, especially fluorination, continues to be a strategy to improve oral bioavailability [133]. Altogether, the unprecedented advances of 2025 reaffirm halogen-containing small-molecule drugs as a cornerstone of modern therapeutics, underscoring their enduring value in innovation, precision design, and transformative clinical impact.

## 5. Conclusions

Halogenation remains one of the most powerful and strategically refined tools in modern medicinal chemistry. As highlighted throughout this review, the deliberate incorporation of fluorine, chlorine, bromine, and, more selectively, iodine continues to shape the physicochemical, pharmacokinetic, and pharmacodynamic profiles of contemporary drug candidates. Fluorine, in particular, has proven indispensable for fine-tuning lipophilicity, metabolic resistance, and target binding through electronic modulation and participation in unique noncovalent interactions. Chlorine further expands structural diversity by enhancing hydrophobic contacts, influencing molecular conformation, and optimizing pharmacokinetic behavior.

Recent FDA approvals in 2025 reinforce the enduring relevance of halogen chemistry in addressing complex therapeutic challenges across oncology, infectious diseases, and central nervous system disorders. Structure–activity relationship (SAR) analyses consistently demonstrate that strategic halogen placement can dramatically alter potency, selectivity, and safety profiles. Advances in synthetic methodologies have also improved the efficiency, regioselectivity, and scalability of halogen incorporation, supporting practical drug development with favorable yields and sustainable approaches.

Beyond traditional electronic and lipophilic effects, growing structural and biophysical evidence underscores the importance of halogen bonding and other directional interactions in stabilizing ligand–target complexes. These insights deepen our understanding of molecular recognition and provide a rational framework for future design strategies.

## Figures and Tables

**Table 1:** Names of the discussed halogenated drugs (FDA-approved in 2025) with additional data, including their therapeutic indication.

Drug Name	Company Name	Physicochemical Properties	Brand Name	Therapeutic Indication
Suzetrigine	VX-548	LogP: 2.42 tPSA: 103.0 LogS: −5.021	Journavx^®^	Treatment of moderate-to-severe pain
Mirdametinib	PD-0325901	LogP: 3.61 tPSA: 90.82 LogS: −5.173	Gomekli^®^	For adult and pediatric patients 2 years of age and older with neurofibromatosis type 1 (NF1)
Avutometinib and defactinib	CH5126766 or RO5126766 (avutometinib); VS-6063 (defactinib)	LogP: 1.91 tPSA: 130.81 LogS: −5.347 LogP: n/a tPSA: 139.98 LogS: −7.014	Avmapki^®^ Fakzynja^®^ Co-Pack	KRAS-mutated recurrent low-grade serous ovarian cancer (LGSOC) after prior systemic therapy
Taletrectinib	DS-6051	LogP: 3.95 tPSA: 75.24 LogS: −5.467	Ibtrozi^®^	Target ROS1 fusion-positive and NTRK fusion-positive non-small cell lung cancer (NSCLC), including brain metastases and resistance mutations
Sunvozertinib	DZD-9008	LogP: 4.53 tPSA: 113.82 LogS: −7.366	Zegfrovy^®^	For patients with advanced or metastatic NSCLC with EGFR exon 20 insertions, post-platinum chemotherapy
Sebetralstat	KVD-900	LogP: 2.19 tPSA: 95.83 LogS: −4.422	Ekterly^®^	For the on-demand treatment of hereditary angioedema (HAE) attacks
Rilzabrutinib	PRN-1008	LogP: 3.78 tPSA: 135.38 LogS: −7.724	Wayrilz^®^	For adults with persistent or chronic immune thrombocytopenia (ITP) who have had an insufficient response to a previous treatment
Imlunestrant	LY-3484356	LogP: 5.56 tPSA: 54.29 LogS: −7.553	Inluriyo^®^	For adults with ER-positive, HER2-negative, ESR1-mutated advanced or metastatic breast cancer who had progressed after at least one line of endocrine therapy
Paltusotine	CRN00808	LogP: 4.76 tPSA: 85.64 LogS: −7.627	Palsonify^®^	For the treatment of acromegaly in people who had an insufficient response to surgery and/or for whom surgery is not an option
Remibrutinib	LOU064	LogP: 4.28 tPSA: 109.38 LogS: −6.127	Rhapsido^®^	To treat chronic spontaneous urticaria in adults who remain symptomatic despite H1 antihistamine treatment
Nerandomilast	BI 1015550	LogP: 1.85 tPSA: 102.01 LogS: −3.99	Jascayd^®^	Treatment of adults with idiopathic pulmonary fibrosis (IPF)
Elinzanetant	BAY 3427080	LogP: 6.92 tPSA: 68.61 LogS: −8.461	Lynkuet^®^	Treatment of moderate-to-severe vasomotor symptoms due to menopause
Ziftomenib	KO-539	LogP: n/a tPSA: 119.67 LogS: −8.434	Komzifti^®^	Treatment of adults with relapsed or refractory acute myeloid leukemia with a susceptible nucleophosmin 1 mutation who have no satisfactory alternative treatment options
Sevabertinib	BAY 2927088	LogP: 1,11 tPSA: 102.44 LogS: −5.21	Hyrnuo^®^	For patients with metastatic or locally advanced non-squamous non-small cell lung cancer (NSCLC) whose tumors include activating mutations in the HER2 (ERBB2) tyrosine kinase domain (TKD)
Zoliflodacin	AZD0914	LogP: 1.14 tPSA: 138.87 LogS: −4.792	Nuzolvence^®^	To treat uncomplicated urogenital gonorrhea due to Neisseria gonorrhoeae
Tradipitant	LY686017 or VLY-686	LogP: 6.9 tPSA: 69.75 LogS: −9.479	NEREUS^®^	For the acute prevention of vomiting induced by motion

## Data Availability

The data presented in this study are openly available at https://www.fda.gov/drugs/novel-drug-approvals-fda/novel-drug-approvals-2025 (accessed on 15 January 2025).

## Abbreviations

The following abbreviations are used in this manuscript:

ACN	acetonitrile
AcOH	acetic acid
ADC	antibody–drug conjugate
BBB	blood–brain barrier
BBr3	boron tribromide
BOC	tert-butoxycarbonyl
BTK	Bruton’s tyrosine kinase
BuLi	n-butyllithium
BuOH	n-butanol
CDI	carbonyldiimidazole
CNS	central nervous system
COVID-19	coronavirus disease 2019
Cryo-EM	cryogenic electron microscopy
CPME	cyclopentyl methyl ether
Cs2CO3	cesium carbonate
DBU	1,8-diazabicyclo [5.4.0]undec-7-ene
DCM	dichloromethane
DIAD	diisopropyl azodicarboxylate
DIBALH	diisobutylaluminum hydride
DIEA	diisopropylethylamine
DME	dimethoxyethane
DMF	dimethylformamide
DMSO	dimethyl sulfoxide
EGFR	epidermal growth factor receptor
FAK	focal adhesion kinase
FDA	Food and Drug Administration
GI	gastrointestinal
HATU	O-(7-azabenzotriazol-1-yl)-N,N,N’,N’-tetramethyluronium hexafluorophosphate
HCl	hydrochloric acid
HER2	human epidermal growth factor receptor 2
iPrOH	isopropanol
ITP	immune thrombocytopenia
MAPK	mitogen-activated protein kinase
MCF7	Michigan Cancer Foundation-7 (breast cancer cell line)
MEK	mitogen-activated protein kinase kinase
MeOH	methanol
MeSO2Cl	methanesulfonyl chloride
NCS	N-chlorosuccinimide
NF1	neurofibromatosis type 1
NIS	N-iodosuccinimide
NSCLC	non-small cell lung cancer
PD	pharmacodynamics
PK	pharmacokinetics
PPh3	triphenylphosphine
SFC	supercritical fluid chromatography
SNAr	nucleophilic aromatic substitution
SOCl2	thionyl chloride
TFA	trifluoroacetic acid
THF	tetrahydrofuran
TKD	tyrosine kinase domain
TKI	tyrosine kinase inhibitor
TMSCl	trimethylsilyl chloride
TNBC	triple-negative breast cancer
tPSA	total polar surface area
TsOH	p-toluenesulfonic acid
